# Inhaled ciprofloxacin-loaded poly(2-ethyl-2-oxazoline) nanoparticles from dry powder inhaler formulation for the potential treatment of lower respiratory tract infections

**DOI:** 10.1371/journal.pone.0261720

**Published:** 2021-12-23

**Authors:** Mohammad Zaidur Rahman Sabuj, Tim R. Dargaville, Lisa Nissen, Nazrul Islam

**Affiliations:** 1 Faculty of Health, Pharmacy Discipline, School of Clinical Sciences, Queensland University of Technology, Brisbane, Queensland, Australia; 2 Faculty of Science, School of Chemistry and Physics, Queensland University of Technology, Brisbane, Queensland, Australia; 3 Centre for Immunology and Infection Control (CIIC), Queensland University of Technology, Brisbane, Queensland, Australia; St. John’s University, UNITED STATES

## Abstract

Lower respiratory tract infections (LRTIs) are one of the fatal diseases of the lungs that have severe impacts on public health and the global economy. The currently available antibiotics administered orally for the treatment of LRTIs need high doses with frequent administration and cause dose-related adverse effects. To overcome this problem, we investigated the development of ciprofloxacin (CIP) loaded poly(2-ethyl-2-oxazoline) (PEtOx) nanoparticles (NPs) for potential pulmonary delivery from dry powder inhaler (DPI) formulations against LRTIs. NPs were prepared using a straightforward co-assembly reaction carried out by the intermolecular hydrogen bonding among PEtOx, tannic acid (TA), and CIP. The prepared NPs were characterized by scanning electron microscopy (SEM), dynamic light scattering (DLS), Fourier transform infrared spectroscopy (FTIR), powder X-ray diffraction analysis (PXRD), differential scanning calorimetry (DSC), and thermogravimetric analysis (TGA). The CIP was determined by validated HPLC and UV spectrophotometry methods. The CIP loading into the PEtOx was between 21–67% and increased loading was observed with the increasing concentration of CIP. The NP sizes of PEtOx with or without drug loading were between 196–350 nm and increased with increasing drug loading. The *in vitro* CIP release showed the maximum cumulative release of about 78% in 168 h with a burst release of 50% in the first 12 h. The kinetics of CIP release from NPs followed non-Fickian or anomalous transport thus suggesting the drug release was regulated by both diffusion and polymer degradation. The *in vitro* aerosolization study carried out using a Twin Stage Impinger (TSI) at 60 L/min air flow showed the fine particle fraction (FPF) between 34.4% and 40.8%. The FPF was increased with increased drug loading. The outcome of this study revealed the potential of the polymer PEtOx as a carrier for developing CIP-loaded PEtOx NPs as DPI formulation for pulmonary delivery against LRTIs.

## 1. Introduction

Lower respiratory tract infections (LRTIs) are one of the fatal diseases of the lungs that have severe impacts on global health and the economy. It affects the airways especially the lower regions of the respiratory tracts including the trachea and alveolar sacs. Several infectious gram-positive and negative bacteria including *Streptococcus pneumonia and Pseudomonas aeruginosa* are the primary causative agents of LRTIs. Moreover, this infectious disease is usually associated with other lung-related disorders including cystic fibrosis (CF) and chronic obstructive pulmonary disease (COPD) [1]. In most cases, antibiotics are prescribed to treat the LRTIs [2]. However, both oral and parental forms of antibiotics are given in high doses which need to be administered frequently and cause dose-related adverse effects [3]. Additionally, the global economy is significantly impacted by the current cost associated with the management system of LRTIs [4]. On the contrary, pulmonary drug delivery provides the opportunity to deposit drugs directly into the deep lungs to produce therapeutic benefits at a very low dose of drugs [5]. The lung as an excellent route for systemic delivery of various drugs has some unique features including, high permeability, a good amount of blood supply, and a large surface area (140 m^2^) [6]. Thus, lung delivery is a promising alternative of therapeutics which could provide rapid action for both systemic and local effects [7]. Controlled release of drug using drug-loaded polymer nanoparticles (NPs) has shown promising effects over conventional drug delivery systems including, extended duration of action, enhanced therapeutic effects, limited dose-related complications, and increased patience compliance [5]. The polymers provide some added benefits to use as potential drug carriers including ease formulation characteristics, manageable particle sizes, protecting the formulated drugs from degradation in unfavourable environments [8]. Both synthetic and natural polymers have been investigated for lung delivery and showed benefits over drug alone formulations including prolonged drug release, enhanced dispersibility, improved cellular uptake, and bioavailability [5]. Therefore, scientists have prioritised their research in search of new polymers to introduce them in lung delivery and get better therapeutic effects.

The selection of ciprofloxacin (CIP) as a model drug against LRTIs was done based on its excellent performance against respiratory pathogens including *Staphylococcus aureus*, *P*. *aeruginosa*, and *S*. *pneumonia*. CIP is a fluoroquinolone antibiotic that possess a comprehensive range of antimicrobial activity against most respiratory pathogens especially *P*. *aeruginosa*, which is resistant to most antibiotics. Current delivery methods of CIP are only available in oral and injectable forms for lung diseases [9]. The available doses of CIP against LRTIs vary from 500–750 mg tablets as oral delivery and 400 mg as intravenous delivery. However, oral delivery of CIP for the treatment of LRTIs is compromised by its poor water solubility [10] leading to poor penetration in the lung parenchyma. The bioavailability of CIP from oral delivery is 69% as it distributes into other tissues [11]. Both oral and intravenous administration of CIP has a relatively poor therapeutic effect against LRTIs. To overcome these limitations, the pulmonary delivery of CIP could be a promising alternative for direct delivery into the deep lungs for the management of LRTIs.

Polymers as carriers of inhalable CIP formulations could increase the therapeutic effects of the formulated drugs by delivering them directly into the lungs and providing a controlled release profile [5]. For instance, Günday Türeli et al. [12] formulated CIP-loaded PLGA NPs for lung delivery and found enhanced antimicrobial activity compared to that of the conventional CIP doses with no toxic effects on lung cells. The enhanced antimicrobial activity was also reported by Liu et al. [13] when CIP was formulated with selenium lipid nanocarriers against interstitial lung diseases. Both the reports confirmed that CIP formulations either with polymer or lipid nanocarriers are safe for lung delivery with increased antimicrobial activities. Therefore, currently available high-dose CIP urges the innovation of a low-dose pulmonary CIP formulation in conjugation with polymer NPs for the management of LRTIs.

Recently, poly(2-ethyl-2-oxazoline) (PEtOx) has drawn attention from drug delivery experts because of its potential biological and chemical characteristics [14]. PEtOx has several advantageous features as a drug carrier including high water solubility, low toxicity, good stability, and excellent biocompatibility [15]. Besides, it adds benefits showing great potential for applications in the biomedical field as it presents similar properties like poly (ethylene glycol)s (PEG), such as biocompatibility, protein repellency, and stealth behaviour against mammalian immune system [16]. Moreover, PEtOx with two-pseudo peptides has drawn attention as an alternative to PEG with altered stability against oxidative degradation [17]. Recently, Zhang et al. [18] formulated curcumin-loaded PEtOx-polylactic acid nano-micelles for effective delivery of curcumin and found the enhanced release of the drug from the micelles with increased inhibition effect on cancer cells. Doxorubicin-loaded polyurethane-PEtOx micelles provided a rapid release of the drug by complete disassembly of the micelles. Although blank micelles showed nontoxic effects, doxorubicin-loaded micelles demonstrated cytotoxicity against the cancer cells in this study [19]. Similar findings were also reported by Gao et al. [20] when doxorubicin and bortezomib-loaded PEtOx nanocapsules were used on cancer cells to observe their synergistic effects. Interestingly, PEtOx has never been used for lung delivery to optimize their suitability as a carrier of antibiotics especially for deep lung delivery to treat LRTIs. Literature review reveals that PEtOx has never been used as a nanocarrier for CIP in pulmonary delivery in form of dry powder inhaler (DPI) formulations.

The pulmonary delivery of antibiotics for lung infections has the potential to treat the disease at a low dose with reduced dose-related adverse effects. In this study, a synthetic biocompatible PEtOx polymer in conjunction with tannic acid (TA) was used as a carrier to develop CIP-loaded PEtOx NPs as DPI formulations for lung delivery against LRTIs. To date, the PEtOx has never been investigated in lung drug delivery, and thus a detailed characterization of the developed CIP-loaded PEtOx NPs as DPI formulation has been performed to determine their suitability in pulmonary drug delivery.

## 2. Materials and methods

### 2.1 Materials

PEtOx with MW 50 kDa and polydispersity index (PDI) 3–4; analytical grade TA as the assembler for the preparation of NPs with MW 1701 Da and CIP powder (assay: ≥ 98.0%) were purchased from Sigma-Aldrich, Australia. Analytical grade solvents and reagents were used for all the experimentations.

### 2.2 Methods

#### 2.2.1 Blank PEtOx NPs preparation

Blank PEtOx NPs were prepared by a modified coassembly reaction between the neutral PEtOx polymer and TA [8]. To assemble blank PEtOx NPs, PEtOx was dissolved in 1% w/v deionized water using ultrasonication for 5 minutes to obtain 20 mL PEtOx solution. Meanwhile, TA was dissolved in 0.03% w/v deionized water using ultrasonication for 2 minutes to obtain 20 mL TA solution. Then, TA solution was added dropwise into the PEtOx solution over a magnetic stirrer (Heidolph MR Hei-Standard, Germany) at 1000 rpm. The mixture immediately turned turbid thus indicated the formation of PEtOx/TA assemblies. The formulated NPs were separated from the supernatant by centrifugation at 6,000 rpm (TG16-WS, China) for 20 minutes. Finally, the blank PEtOx NPs were obtained by freeze-drying (Freeze Dryer Alpha 1–4 LD plus, USA) at -80°C.

#### 2.2.2 CIP-loaded PEtOx NPs preparation

For preparing CIP-loaded PEtOx NPs, three different concentrations (i.e., 0.025%, 0.05% and 0.075%) of CIP were added into 20 mL of TA solution (0.03% w/v TA solution in deionized water) and another two concentrations (i.e., 0.1% and 0.125%) of CIP were added into 20 mL of TA solution (0.06% w/v TA solution in deionized water). These were done to compare the effects of various doses of CIP in the prepared NPs which gave 5 mg, 10 mg, 15 mg, 20 mg, and 25 mg CIP in weights in each batch respectively. The compositions of all the formulations are summarized in Table 1. All other conditions were kept the same as above.

**Table 1:** Compositions of the NPs prepared in this study.

Formulation	PEtOx (mg)	TA (mg)	CIP (mg)
**Blank PEtOx**	200	6	-
**5 mg CIP-PEtOx**	200	6	5
**10 mg CIP-PEtOx**	200	6	10
**15 mg CIP-PEtOx**	200	6	15
**20 mg CIP-PEtOx**	200	12	20
**25 mg CIP-PEtOx**	200	12	25

**Fig 1:**
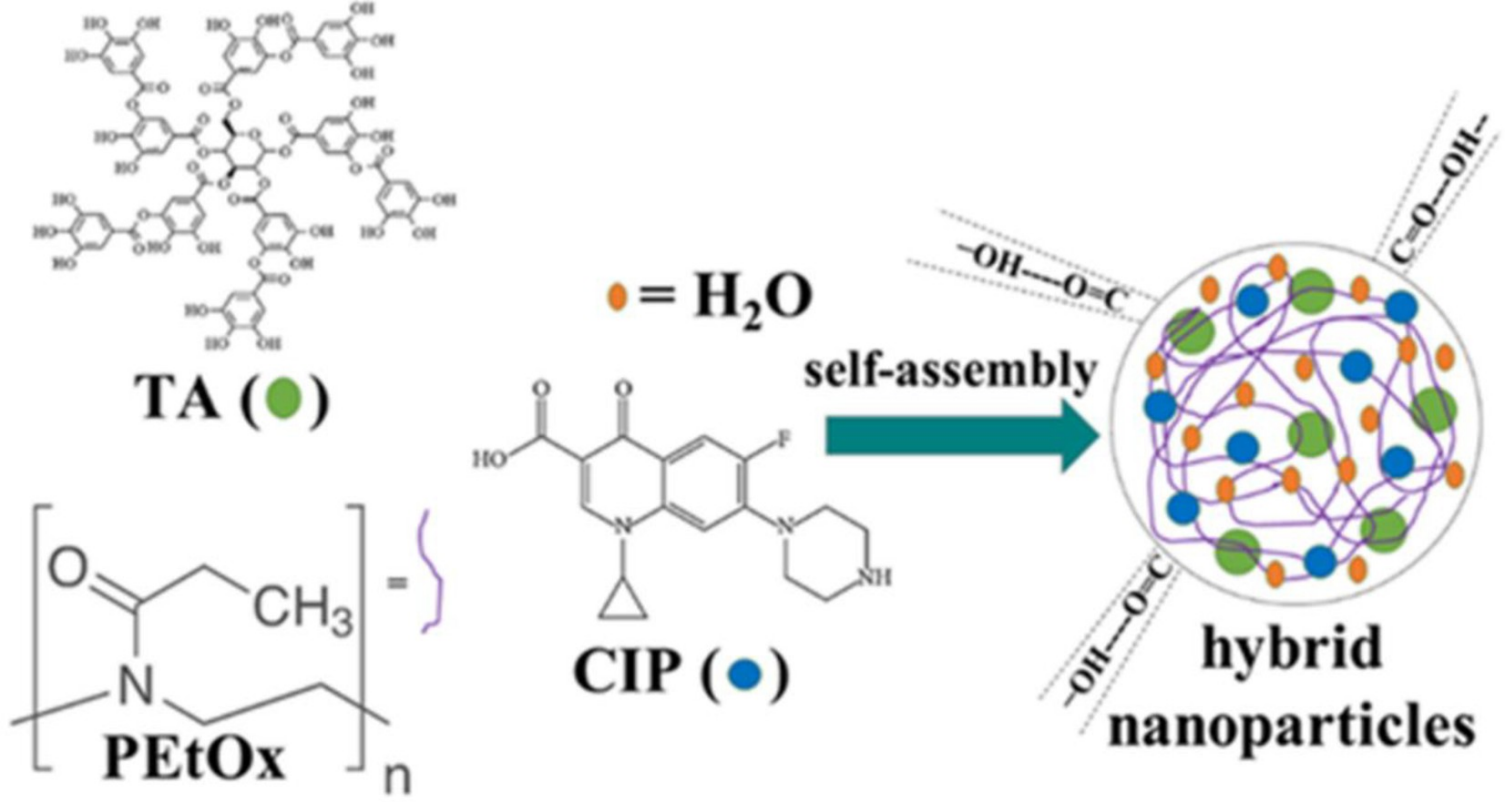
Chemical structure of poly(2-ethyl-2-oxazoline) (PEtOx), ciprofloxacin (CIP), and tannic acid (TA) and the simplified demonstration of nanoparticles (NPs) formation in water.

A schematic diagram of the assembly reactions for the formation of NPs is demonstrated in Fig 1.

#### 2.2.3 Morphology analysis by scanning electron microscopy (SEM)

Using SEM (Tescan Mira3 Brno, Czech Republic), the morphology of the NPs was examined. The particle size of NPs was measured using an ImageJ software before and after freeze-drying. Before freeze-drying, a drop (10 μL) of the blank and CIP-loaded PEtOx NPs suspensions were placed onto the silicon wafer and air-dried. The freeze-dried blank and CIP-loaded PEtOx NPs powder were mounted separately onto the surface of aluminium stub with a carbon adhesive tape. Then the access particles were removed from the adhesive tape by blowing nitrogen gas. Both samples were coated with a conductive layer of sputtered gold (argon gas pressure of 0.5 mbar, current of 30 mA, and coating time of 75 s). The prepared samples were observed under high vacuum secondary electron images with a stepping-up voltage of 5 kV. The working distance was kept at 8.08 mm for this study.

#### 2.2.4 Particle size and size distribution measurement

Blank PEtOx NPs and CIP-loaded PEtOx NPs were studied by a Zetasizer Nano ZS 90 (Malvern Instruments, UK) to determine their particle size and size distribution or polydispersity index (PDI). Particle size and PDI of the NPs were analysed before and after freeze-drying to understand the effects of freeze-drying on the prepared particles. The suspensions of the NPs were ultra-sonicated for 5 minutes before freeze-drying. The freeze-dried powder (2 mg) was suspended in 5 mL deionized water by ultrasonication for 12 minutes before the analysis. Size measurements of the NPs were carried out at a scattering angle of 90°. Malvern Zetasizer Software was used to analysis the particle size and PDI. Each sample was measured in triplicate. The mean diameter ± SD was reported as the particle size and PDI.

#### 2.2.5 Surface charge or zeta potential

The zeta potential of the prepared NPs was confirmed using the same Zetasizer Nano ZS 90 (Malvern Instruments, UK) that was used for analysing the particle size and PDI. As demonstrated in the previous section, the suspensions of the NPs were ultra-sonicated for 5 minutes before freeze-drying. Meanwhile, the freeze-dried powder (5 mg) was dispersed in 10 mL deionized water by ultrasonication for 12 minutes before measuring the zeta potential of the NPs. Each sample was measured in triplicate.

#### 2.2.6 Particle density and flow property

Carr’s index (CI), Hausner ratio (HR), and angle of repose (θ) parameters are used to estimate the flow properties of powder samples for drug delivery. Eqs 1 and 2 [21] were used to calculate the bulk density (ρ_b_) and tapped density (ρ_t_) of the prepared DPI formulations.


$$\rho_{b}=\frac{m_{0}}{V_{0}}$$ (1)

$$\rho_{t}=\frac{m_{0}}{V_{1}}$$ (2)

The findings were used to calculate the CI, HR, and θ values. Powder NPs samples were measured by graduated cylinder to determine their ρ_b_ and ρ_t_ in a tapped density tester (ERW-SVM101202, ERWEKA, Germany). 1 ± 0.5 g powder NPs samples (*m*_0_) were filled in a 10 mL graduated cylinder and plain volumes (*V*_0_) were recorded to determine the ρ_b_ (g/mL). Subsequently, the graduated cylinder was fastened with the density tester and 500 mechanical taps were carried out. The new volume (*V*_1_) of the graduated cylinder was recorded to calculate the ρ_t_ (g/mL). Then, *V*_1_ and *V*_0_ were used to calculate the Carr’s index and Hausner ratio by the Eqs 3 and 4 below [22]. Each sample was measured in triplicate.


$$CI=\frac{100(V_{0}-V_{1})}{V_{0}}$$ (3)

$$HR=\frac{\rho_{t}}{\rho_{b}}$$ (4)

The angle of repose was calculated using Eq 5 [22] below.


$$\theta =\tan^{-1}(\frac{2h}{d})$$ (5)

The angle of repose is a comprehensive variable to determine the powder flow properties of the NPs. It is the steepest angle to the horizontal plane and the cone-like pile of granules. To measure the angle of repose the NPs powder samples (250 ± 0.5 mg) were poured through a funnel (18 mm diameter, 50 mm height, and 2 mm orifice) and retained into a 5 mL beaker (10 mm diameter) which was placed approximately 3 cm below the funnel end. Then, the height (h) and cone diameter (d) established by the particles were evaluated using Eq 5.

#### 2.2.7 Differential scanning calorimetry (DSC)

CIP alone, blank PEtOx NPs, and CIP-loaded PEtOx NPs were studied to understand their thermal behaviour by a DSC instrument (TA Instruments DSC; model Q100). Lyophilized powder samples (2 ± 0.1 mg) were precisely weighed and placed in a hermetically sealed aluminium pan. Then, the samples were scanned from 20°C to 300°C at a heating rate of 10°C/min. A customized empty sealed pan was examined in the same way to use it as a reference. Each sample was examined in triplicate.

#### 2.2.8 Thermogravimetric analysis (TGA)

The calorimetric behaviour and decomposition of CIP alone, blank PEtOx NPs, and CIP-loaded PEtOx NPs were examined by a TGA instrument (NETZCCH STA 449F3; Selb, Germany), by measuring the mass loss. The lyophilized powder (5–8 mg) was placed in a customized aluminium pan, sealed hermetically, and scanned from 20°C to 1000°C at the heating rate of 10°C/min. An empty customized, hermetically sealed pan was examined in the same way to use it as a reference. Each sample was examined in triplicate.

#### 2.2.9 Crystallinity: Powder X-ray diffraction (PXRD)

The crystallinity of CIP alone and the five batches of CIP-loaded PEtOx NPs were collected by powder X-ray diffraction patterns in Debye-Scherrer geometry using Rigaku® SmartLab diffractometer. Operating parameters were used as follows: 40 kV CuKα radiation, 40 mA current, 2θ data collection range of 3–120, and a scan speed of 2.0°/min.

#### 2.2.10 Attenuated total reflection-Fourier transform infrared (ATR-FTIR)

ATR-FTIR spectra of CIP alone, blank PEtOx NPs, and CIP-loaded PEtOx NPs were examined by a Thermo iS50 FTIR spectrometer (Nicolet, Madison, USA) equipped with an angle of incidence of 40° and a deuterated triglycine sulphate (DTGS) detector. A high-pressure clamp was used to secure a small amount of lyophilized powder sample on top of the diamond crystal. Operating parameters were used to collect spectral data are as follows: 8 cm^-1^ resolution, 64 scans, and data collection range of 4000–400 cm^-1^. Data was then analysed utilizing the analytical software OMNIC (Nicolet Instrument Corp., Version 9.2, Madison, WI, USA).

#### 2.2.11 Drug loading

The amount of CIP accumulated either on the surface or in the matrix of the NPs after freeze-drying was calculated by Eq 6.


$$Drug\;loading\;(\%)=\frac{total\;drug-free\;drug}{particle\;weight}\times 100$$ (6)

Eq 7 was used to determine the entrapment efficiency depending on the primary amount of CIP used for NPs formulation and the amount of CIP assembled within the NPs after lyophilization.


$$Entrapment\;efficiency\;(\%)=\frac{total\;drug-free\;drug}{total\;drug}\times 100$$ (7)

Primarily the liquid NPs were centrifuged, and the free drugs were separated as supernatant. Then, the supernatant was mixed with an excess amount of 0.1 N HCl (1:9) solution and analysed by UV spectrophotometry (Cary 60 UV-Vis Spectrophotometer, USA). 275 nm wavelength was used to detect CIP as the maximum absorbance was found in this wavelength. Then, the amount of the remaining CIP in the supernatant was deducted from the primary amount of CIP which was used for formulating the NPs. Each sample was examined in triplicate.

#### 2.2.12 *In vitro* controlled release study

*In vitro* CIP release from the CIP-loaded PEtOx NPs was performed in phosphate-buffered saline (PBS) (pH 7.4 ± 0.2) followed by a previously reported method with little modifications [21]. Briefly, CIP release from the NPs was studied in a temperature-controlled dialysis membrane bag. The concentration of the released CIP was determined by a UV-visible spectrophotometer. The dialysis membrane bag (MW 12,000 Da, Sigma-Aldrich, USA) was kept in deionized water for 24 hours to make it suitable for the release study. 3 mg CIP-loaded PEtOx NPs were dispersed in 3 mL PBS by ultrasonication for 5 minutes and were mounted into the dialysis bag which was then placed into a 100 mL beaker. A small stirrer magnetic bar was put into the acceptor compartment to keep the mixing uniform while conducting the drug release study. A magnetic stirrer was used to keep the gentle stirring speed at 100 rpm and a constant temperature of 37°C.

At zero time, the prepared solution was placed into the donor compartment (dialysis bag) and the donor compartment was placed into the acceptor compartment (beaker) having 50 mL PBS. Throughout the experimentation, sink condition was maintained. At fixed time intervals 5 mL of the release samples were withdrawn from the acceptor compartment of the drug release chamber and immediately equal volume freshly prepared PBS solution was added into the system. The withdrawn samples were analysed further with UV spectrophotometry to determine the release concentration of CIP from the CIP-loaded PEtOx NPs. Using a UV-visible spectrophotometer, the drug was determined at 275 nm wavelength. The concentration of CIP was determined by a validated standard curve (S2 Fig in S1 File). The drug release experiment was continued for 14 days to ensure maximum release. Each measurement was conducted three times (n = 3).

The release study of CIP alone was also performed using the same dialysis membrane to determine the time taken by CIP to completely release from the bag. As indicated before, a 3 mg CIP only powder was dispersed in 3 mL PBS by ultrasonication for 5 minutes and mounted in the dialysis bag. In this experiment, aliquots were withdrawn at every 15 minutes for the first 2 hours and every 2 hours in 24 hours and the concentration of CIP was measured by the same UV-visible spectrophotometry method. The cumulative release profiles of CIP from CIP-loaded PEtOx NPs were determined by preparing a plot between the cumulative percentage of CIP release versus time.

#### 2.2.13 Kinetics of drug release

There are five commonly used mathematical models to determine the drug release mechanism. CIP release data from the CIP-loaded PEtOx NPs were fitted into those models to understand the release mechanism of CIP from PEtOx polymer. These models are known as zero-order model, first-order model, Higuchi’s square-root model [23], Korsmeyer-Peppas model [24], and Hixson-Crowell model [25] and they are expressed by the following equations:

$$zero‐order\;model:\;F=k_{0}t$$ (8)

$$first‐order\;model:\;In\;(1-F)=-k_{1}t$$ (9)

$$Higuchi\;model:\;F=K_{H}t^{\frac{1}{2}}$$ (10)

$$Korsmeyer‐Peppas\;model:\;F=k_{K-P}t^{n}$$ (11)

$$Hixson‐Crowell\;model:\;1-{\left(1-F\right)}^{\frac{1}{3}}=k_{\frac{1}{3}}t$$ (12)

Where,

*F* = cumulative fraction of drug release at time t

*n* = Korsmeyer-Peppas model release exponent

*k*_*0*_, *k*_*1*_, *k*_*H*_, *k*_*1/3*_, *k*_*K-P*_ = Apparent release rate constants of the respective mathematical models.

#### 2.2.14 *In vitro* aerosolization study

A twin-stage impinger (TSI, Apparatus A; British Pharmacopoeia, 2000) was used to determine the aerosolization performance of the prepared powder formulations (CIP alone, blank PEtOx NPs, and CIP-loaded PEtOx NPs) [7]. A capsule-based DPI device Breezhaler^®^ (Novartis Pharmaceuticals Pvt Ltd, NSW, Australia) was used in this experiment. 7 mL and 30 mL PBS (pH 7.4 ± 0.2) (as solvent) was dispended in Stage 1 (S1) and Stage 2 (S2) of the TSI, respectively. A vacuum pump (D-63150, Erweka, Germany) was used to draw an airflow rate to 60 ± 5 L/min at the mouthpiece. The airflow rate was controlled by a calibrated digital flow meter (Fisher and Porter, Model 10A3567SAX, UK).

The dispersibility performance of the prepared NPs was determined following the recommended method of British Pharmacopoeia [26]. Hard gelatine capsules (size 3; Fawns and McAllan Pty., Australia) were used to perform the aerosolization studies. The formulated NPs powder samples were inserted (20 ± 1 mg) in the capsules and eventually were inserted in the Breezhaler^®^. The capsules were twisted with the aid of the Breezhaler^®^ and aerosolized at 60 ± 5 L/min for 5 s to disperse the DPI formulations within the three stages of the TSI device. The cut-off diameter of S2 was 6.4 μm. Upon completion of each experiment, all the stages of the TSI device were washed separately by PBS and the quantity of CIP was evaluated by both HPLC assay and gravimetric analysis [27].

The gravimetric analysis of the washings was performed by a validated method developed in our lab [27]. A pre-dried and pre-weighed filter paper (orifice 0.20 μm, Phenomenex, USA) was used to filter the collected washings from each stage of the TSI device. Continuous filtration was carried out to get clear washing solutions. The particles accumulated on the surface of the filter paper were dried at 60°C for 24 hours. Completely dried filter paper provided a constant weight, and the mass was used to determine the amount of NPs gravimetrically. The filtrated solutions of the CIP-loaded PEtOx NPs were collected and analysed further by the HPLC method to determine the concentration of CIP in PBS solution. A standard plot was developed using peak height versus concentration of CIP in PBS. This standard plot was used to determine the concentration of CIP in each stage of the TSI device. Finally, the mass of CIP was added with the concentration of CIP to obtain the total experimental data. Hence, both gravimetric and analytic methods were used to determine the deposited CIP into stage 2 from CIP-loaded PEtOx NPs.

NPs size less than 200 nm could easily pass through the orifice of 0.2 μm filter paper, however, powder NPs are likely to agglomerate and eventually block the orifices of the filter paper upon repeated filtration. Thus, the gravimetric analysis also provided the amount of particle depositions in several stages of the TSI device. Four parameters were used to determine the particle depositions of the formulated NPs in all the stages of the inhaler device including, recovered dose (RD), emitted dose (ED), fine particle fraction (FPF), and fine particle dose (FPD). These parameters were determined by using Eqs 13 and 14 below. Blank PEtOx NPs were only analysed gravimetrically, while CIP-loaded PEtOx NPs were analysed by both gravimetric and analytic methods.


$$ED=\frac{S1+S2}{RD}\times 100$$ (13)

$$FPF=\frac{S2}{RD}\times 100$$ (14)

RD was determined as the percentage of the total amount of drug collected from Breezhaler^®^, S1, and S2; while ED was the percentage of drug emitted into S1 and S2 of the TSI. FPF is the respirable fraction of the formulated NPs and the percentage of particles deposited into S2 of the TSI device out of RD. Before analysing the CIP-loaded PEtOx NPs filtrated aliquots in HPLC it was kept in gentle stirring (100 rpm) at 37°C for 7 days to allow sufficient time to release the loaded CIP from the NPs.

#### 2.2.15 HPLC assay to determine CIP analytically

CIP was analysed by HPLC using a previously reported method with little modifications [28]. An Agilent HPLC Series 1100 with Autosampler and Diode Array Detector (Atlas) Hewlett-Packard (Waldbronn, Germany), was used to carry out the analytical experiment. An Agilent Poroshell 120 EC-C18 4 μm column (4.6 × 250 mm) (USA) was used as the stationary phase. The mobile phase composition was 80:20 acetonitrile:0.1% trifluoracetic acid (pH 2.0). The parameters were used for the experiment are as follows: 10 μL injection of each sample by the autosampler, flow rate 1 mL/min, total run time 10 min. CIP was detected by UV absorbance at 275 nm wavelength. The findings were used to prepare a calibration curve of CIP in PBS at concentrations ranging from 1 μg/mL to 100 μg/mL with the limit of quantification (LOQ) 1 μg/mL (S3 Fig in S1 File). Online ChemStation software was used to operate the HPLC assay.

### 2.3 Statistical analysis

Statistical analysis was completed by applying a one-way analysis of variance (ANOVA) with Bonferroni multiple-comparison test. P values of *p* < 0.05 were considered as a significant difference. All measurements were taken in triplicate and expressed as mean values and standard deviations.

## 3. Results and discussion

### 3.1 Blank PEtOx NPs and CIP-loaded PEtOx NPs preparation

Intermolecular hydrogen bonding among neutral PEtOx, TA, and CIP was used to prepare the blank PEtOx NPs and CIP-loaded PEtOx NPs. A straightforward coassembly reaction was utilized to form the NPs. The coassembly of PEtOx and potential hydrogen bond donor TA were capable to form stable NPs through hydrogen bonding in water [8]. To allow an appropriate assembly, 20 mL PEtOx and TA aqueous solutions were prepared separately. Then, TA aqueous solution was added dropwise under continuous stirring in the PEtOx aqueous solution. In the process of NPs preparation, the stirring speed, and the concentration of both PEtOx and TA in water were important factors to control the particle size, however, size distribution was not completely uniform which is common in powder formulations. The molecular weight of the polymer, concentration, ratio of both PEtOx and TA, and the stirring speed of the mixing are the combined factors that could affect the final size of the particles.

A 0.03% TA solution in 1% PEtOx with a 1:1 volume ratio yielded blank PEtOx NPs sized less than 200 nm while PDI was also less than 1 as presented in Table 2. Several stirring speeds (100–1000 rpm) were used preliminarily to obtain the desired NPs. We selected the fast stirring speed (1000 rpm) in this study to obtain the desired size of the NPs as stirring speed plays important role in the size of the formulated NPs [29]. In this study, the final DPI formulations from the liquid NPs were obtained by lyophilization to keep the formulated samples stable for the long term and maintain acceptable relative humidity [30].

**Table 2:** Particle size, PDI and zeta potential analysis of blank PEtOx NPs and different amounts of CIP-loaded PEtOx NPs (data presented as mean ± S.D., n = 3).

Formulation	Z-average size d before freeze-drying (nm)	Z-average size d after freeze-drying (nm)	PDI before freeze-drying	PDI after freeze-drying	Zeta potential before freeze-drying (-mv)	Zeta potential after freeze-drying (-mv)
**Blank PEtOx**	151.6 ± 1.7	196.7 ± 24.0	0.25 ± 0.1	0.35 ± 0.1	20.2 ± 1.4	30.5 ± 2.0
**5 mg CIP-PEtOx**	159.4 ± 4.5	299.7 ± 18.2	0.19 ± 0.0	0.31 ± 0.0	17.9 ± 0.8	28.1 ± 1.5
**10 mg CIP-PEtOx**	185.6 ± 0.5	317.4 ± 27.6	0.21 ± 0.0	0.31 ± 0.0	14.4 ± 1.7	25.8 ± 1.0
**15 mg CIP-PEtOx**	199.0 ± 1.8	353.0 ± 55.1	0.32 ± 0.0	0.28 ± 0.1	12.9 ± 0.5	22.3 ± 0.7
**20 mg CIP-PEtOx**	189.8 ± 5.3	249.0 ± 52.2	0.29 ± 0.1	0.33 ± 0.1	10.8 ± 1.3	21.2 ± 0.9
**25 mg CIP-PEtOx**	169.9 ± 3.3	200.0 ± 22.0	0.19 ± 0.0	0.33 ± 0.0	9.1 ± 0.2	18.5 ± 0.8

CIP-loaded PEtOx NPs were prepared by the addition of CIP in TA solution at a series of weights from 5–25 mg at two different fixed conditions one was the same as the concentration of the blank PEtOx NPs preparation and another with increased concentration of TA solutions. Before the coassembly of PEtOx and TA aqueous solution with CIP, the assembly behaviour of PEtOx/CIP and TA/CIP was examined. The addition of PEtOx or TA aqueous solution with CIP solution resulted in a clear solution which indicated no NPs formation. Therefore, the coassembly between CIP solution with PEtOx/TA aqueous solution was ruled out. However, dropwise addition of the premixed TA and CIP solution with PEtOx aqueous solution resulted opaque mixture which confirmed the formation of NPs. Thus, the dropwise addition of TA and CIP solution to the PEtOx aqueous solution was able to avoid the dominant assembly between PEtOx and TA [31]. The average diameter of the CIP-loaded PEtOx NPs was likely to increase with the increased concentration of CIP. However, the average particle size tends to decrease with the increasing concentration of TA from 0.03% to 0.06% [8].

### 3.2 Morphology analysis by scanning electron microscopy (SEM)

**Fig 2:**
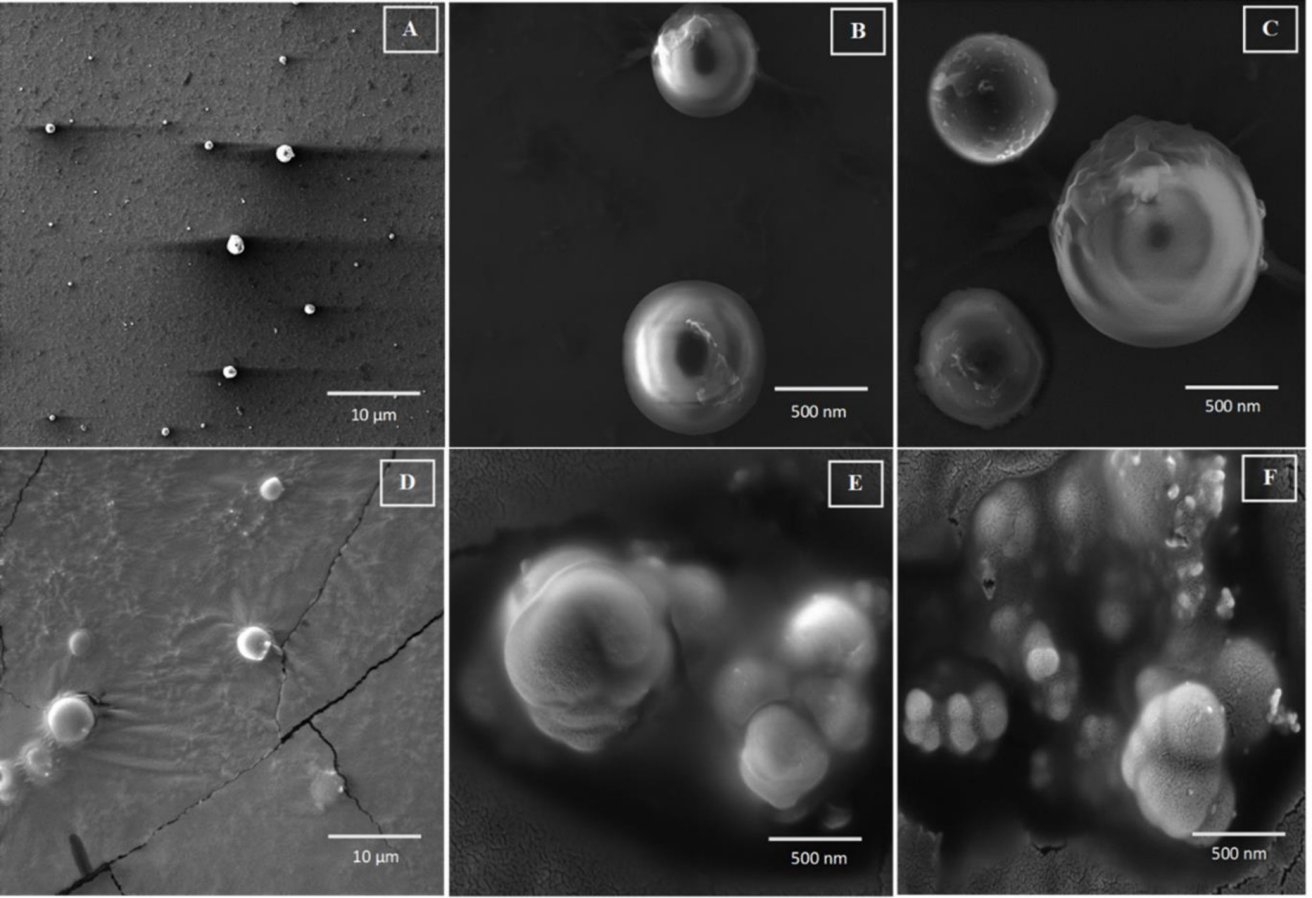
SEM photomicrographs of blank PEtOx NPs and CIP-loaded PEtOx NPs (A) blank PEtOx NPs before freeze-drying; 10 μm (B) 5 mg CIP-loaded NPs before freeze-drying; 500 nm (C) 10 mg CIP-loaded NPs before freeze-drying; 500 nm (D) 15 mg CIP-loaded NPs after freeze-drying; 10 μm (E) 20 mg CIP-loaded NPs after freeze-drying; 500 nm (F) 25 mg CIP-loaded NPs after freeze-drying; 500 nm.

The blank PEtOx NPs and CIP-loaded PEtOx NPs were analysed morphologically by SEM to confirm their size and understand their shape along with the surface properties. This experiment was carried out before and after freeze-drying the NPs. All blank PEtOx NPs showed spherical shapes with smooth surfaces before freeze-drying (Fig 2A). Consequently, CIP-loaded PEtOx NPs showed rough surfaces before and after freeze-drying thus suggesting that a certain amount of CIP was adhered on the surface of the NPs (Fig 2C, 2E and 2F). However, the freeze-dried blank NPs found to be agglomerated with the increased average size (375.6 nm). The particle sizes of the CIP-loaded PEtOx NPs were found between 143–187 nm (Fig 2B and 2C) and 173.5–789.2 nm before and after freeze-drying, respectively. The increased size of the freeze-dried CIP-loaded PEtOx NPs was due to the agglomeration of the NPs. Thus, the freeze-drying process significantly (*p* < 0.05) increased the size of the prepared NPs [32]. However, few individual particles were also observed in SEM images after freeze-drying (Fig 2D). The SEM images confirmed that the agglomerated particles were also within the nano range after freeze-drying.

### 3.3 Particle size and size distribution

**Fig 3:**
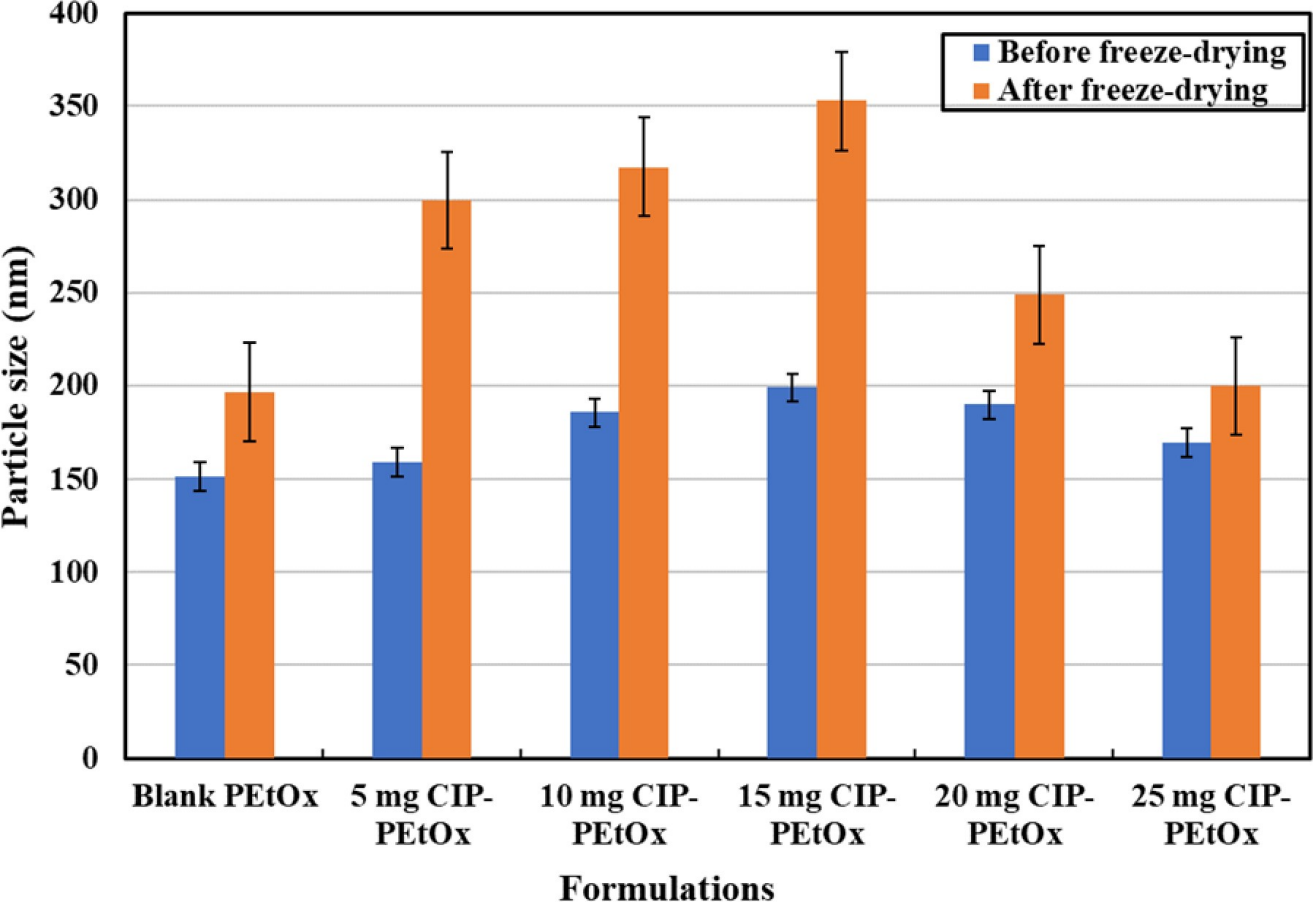
The particle size of the prepared NPs before and after freeze-drying (data presented as mean ± S.D., n = 3).

Average particle size and size distribution or PDI of the blank PEtOx NPs and CIP-loaded PEtOx NPs were presented in Table 2 and Fig 3. Blank PEtOx NPs before freeze-drying demonstrated a mean particle diameter of 151 ± 1.7 nm. However, the freeze-dried blank PEtOx NPs showed a mean diameter of 196.7 ± 23.9 nm, which indicated the increased particle size after freeze-drying because of the formation of agglomerates. The CIP-loaded PEtOx NPs showed similar pattern after freeze drying. The average particle size of CIP-loaded PEtOx NPs before freeze-drying varied between 159.8 to 199.0 nm. While the freeze-dried NPs showed an average particle size between 249.0 to 353.0 nm, which indicated that the sizes of the prepared NPs (blank and CIP-loaded PEtOx) were significantly increased (*p* < 0.05) after freeze drying. The increased particle size is a common phenomenon of freeze-dried products because of the aggregation of the particles during the freeze-drying process [32].

The findings showed that while formulating the CIP-loaded PEtOx NPs increasing CIP concentration at fixed TA concentration resulted in increased particle size. However, mean particle size was decreased with increased TA concentration despite the increasing concentration of CIP. The blank NPs before and after freeze-drying showed bimodal size distributions as demonstrated in S4A and S4B Fig in S1 File. However, CIP-loaded PEtOx NPs showed unimodal size distribution before and after freeze-drying, respectively. DLS size measurements as presented in Table 2 of the NPs were different from the measurements of SEM Fig 2. The measurement mechanisms of DLS and SEM are different. DLS measured the particles in the swollen state whereas SEM measures the particles in the dry collapsed state thus DLS measurement (199.0 nm) revealed a slightly larger diameter than the SEM measurement (186.8 nm). However, powder NPs were resuspended by ultrasonication before measuring them in DLS thus the measurement revealed deagglomerated particle size rather than measuring the aggregation of the NPs measured by SEM. Moreover, both the measurements confirmed that the prepared NPs were within the inhalable size range (the aerodynamic diameter of the inhalation range < 5 μm) [33].

### 3.4 Zeta potential measurement to determine the surface charge

DLS measurement revealed that both before and after freeze-drying the blank PEtOx NPs and CIP-loaded PEtOx NPs were negatively charged. However, increasing the amount of CIP in the NPs decreased the surface charge as presented in Table 2. Freeze-dried powder NPs showed significantly increased (*p* < 0.05) surface charge compared to the surface charge of the NPs before freeze-drying. As NPs were aggregated after freeze-drying and increased the particle size, which led to decrease the surface area of the particles, and thus resulted in decreasing surface charge [34]. A linearity trend (S5A and S5B Fig in S1 File) was observed between the surface charge and the concentration of CIP in NPs, which indicated that the surface charge of the drug loaded NPs decreased with the increasing amount of CIP. Therefore, it can be confirmed that the surface of the NPs is covered with an adequate amount of CIP adhered on the surface of the NPs. It was noted that increased drug-loading resulted in increased surface area or particle size and covered more surface area thus resulted in decreased surface charge as presented in Table 2. CIP-loaded PEtOx NPs showed significantly lower (*p* < 0.05) surface charge compared to blank PEtOx NPs before and after freeze-drying, thus, confirmed that the surface negative charges were covered by the increased amount of CIP on the surface of the NPs [7]. In addition, TA provided low pH during particles formation and CIP usually shows a positive charge at low pH because of the protonation of the amino group [35], thus the negative charge of the PEtOx NPs was covered with the accumulation of increased CIP on the surface of the NPs. Surface charge measurement of the particles is important as it is associated with the dispersibility behaviour of the particles because of the repulsive forces [7]. Particle surface charge could effectively improve the flow properties of the NPs by reducing the cohesion forces among the NPs. Thus, the high surface charge of the prepared NPs could play an important role in increasing the dispersibility behaviour of the prepared NPs [36].

### 3.5 Particle density and flow property

There is a critical relationship between the performance of DPI formulations and the powder flow properties. Table 3 represents the powder flow properties of blank PEtOx NPs and CIP-loaded PEtOx NPs. Blank PEtOx NPs, 5 mg, 10 mg, and 15 mg CIP-loaded PEtOx NPs showed lower bulk and tapped density compared to 20 mg and 25 mg CIP- loaded PEtOx NPs. Drug-loaded NPs formulations showed increased particle density with increased drug loading.

**Table 3:** Physical properties of blank PEtOx NPs and CIP-loaded PEtOx NPs (data presented as mean ± S.D., n = 3).

	Blank PEtOx	5 mg CIP-PEtOx	10 mg CIP-PEtOx	15 mg CIP-PEtOx	20 mg CIP-PEtOx	25 mg CIP-PEtOx
**Bulk density (g/mL)**	0.1 ± 0.0	0.1 ± 0.0	0.1 ± 0.0	0.1 ± 0.0	0.2 ± 0.0	0.2 ± 0.1
**Tapped density (g/mL)**	0.2 ± 0.0	0.2 ± 0.0	0.2 ± 0.0	0.2 ± 0.0	0.2 ± 0.0	0.2 ± 0.0
**CI**	33.5 ± 0.6	31.2 ± 0.6	28.7 ± 1.5	25 ± 0.6	16 ± 0.2	13.4 ± 2.1
**HR**	1.5 ± 0.0	1.5 ± 0.0	1.1 ± 0.0	1.3 ± 0.0	1.2 ± 0.0	1.2 ± 0.1
**θ**	38.6 ± 1.5	34.6 ± 0.8	32.7 ± 0.7	27.4 ± 0.6	27.4 ± 0.2	25.3 ± 0.5

Powder flow property is evaluated by the analysis of CI and HR. A CI value less than 25% is considered as good flowability while greater than 25% is considered as poor flowability indicating the characteristic of cohesive powder. Besides, the HR value of <1.25 indicates good flowability; and a value of >1.25 is considered as poor flowability. However, these values could only provide the estimation of the powder flow properties of the formulated NPs; measuring methodologies could affect the values of these two parameters. The CI values of the lyophilized NPs powders ranged from 13.37% to 33.46% and HR values were between 1.10 and 1.50. The 20–25 mg CIP-loaded PEtOx NPs showed <25% CI while 10 mg, 20 mg, and 25 mg CIP-loaded PEtOx NPs showed <1.25 HR values, indicating 20 mg and 25 mg CIP-loaded PEtOx NPs produce good flow properties in both parameters. However, blank PEtOx and 5 mg CIP-loaded PEtOx NPs showed higher CI and HR values exhibiting poor flowability. Both 20 mg and 25 mg CIP-loaded PEtOx NPs showed promising flow properties, which could be beneficial for the dispersibility behaviour of the DPI formulations.

Table 3 also represents the angle of repose (θ) values of the formulated NPs. The angle of repose could also provide important information on the powder flow properties of the formulated NPs. The findings showed that θ values decreased with the increased amount of CIP within the CIP-loaded PEtOx NPs formulations. Wang et al. [7] reported similar findings while determining the powder flow properties of nicotine hydrogen tartrate-loaded chitosan NPs for DPI formulations.

### 3.6 Differential scanning calorimetry (DSC)

**Fig 4:**
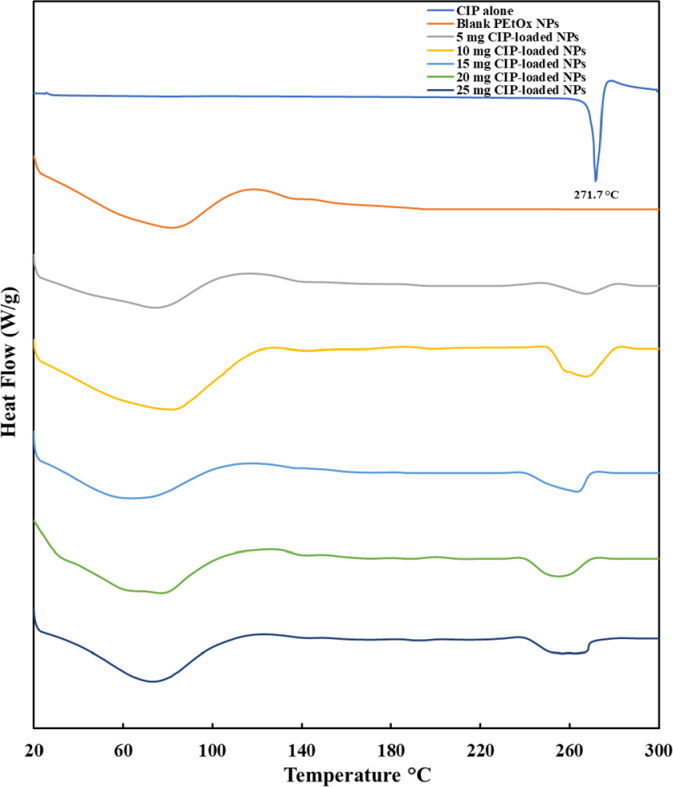
The DSC thermograms of CIP alone, blank PEtOx NPs, and different amounts of CIP-loaded PEtOx NPs (exothermic up).

Fig 4 demonstrates the DSC thermograms of CIP alone, blank PEtOx NPs, and different amounts of CIP-loaded PEtOx NPs. CIP alone demonstrated a sharp characteristic peak at 271.7°C owing to the melting of crystalline nature. Blank PEtOx NPs demonstrated a broad characteristic peak at 65.1°C with a maximum peak point at 82.9°C due to the binding water loss of the blank PEtOx NPs [37]. The broad endotherms from CIP-loaded PEtOx NPs were comparable with the endothermic peak of blank PEtOx NPs and were similar with the binding water loss of the blank PEtOx NPs. CIP-loaded PEtOx NPs showed a minor change in their thermal behaviour, as in most of the cases the melting points of CIP were shifted barely from the original peak of CIP alone. This indicates that there is a possibility of drug-polymer interaction to form the NPs [31]. However, the melting peaks of CIP-loaded PEtOx NPs were in the range of 250–270°C (Fig 4; maximum peaks were in between 260–270°C for various amounts of CIP in the NPs) that indicated the presence of a sufficient amount of CIP in the NPs. This suggested that CIP has been incorporated with the PEtOx NPs and a minor shift of endothermic peaks indicates the interaction between the polymer and CIP. However, the range of melting points is still very close to the CIP alone (271.7°C) thus no changes of the crystal integrity were found within the prepared formulations [37]. The same characteristics of the shifted melting peak for CIP were also reported by Bag et al. [38] when CIP was studied to determine its crystalline characteristics in a drug-drug salt formation.

### 3.7 Thermogravimetric analysis (TGA)

**Fig 5:**
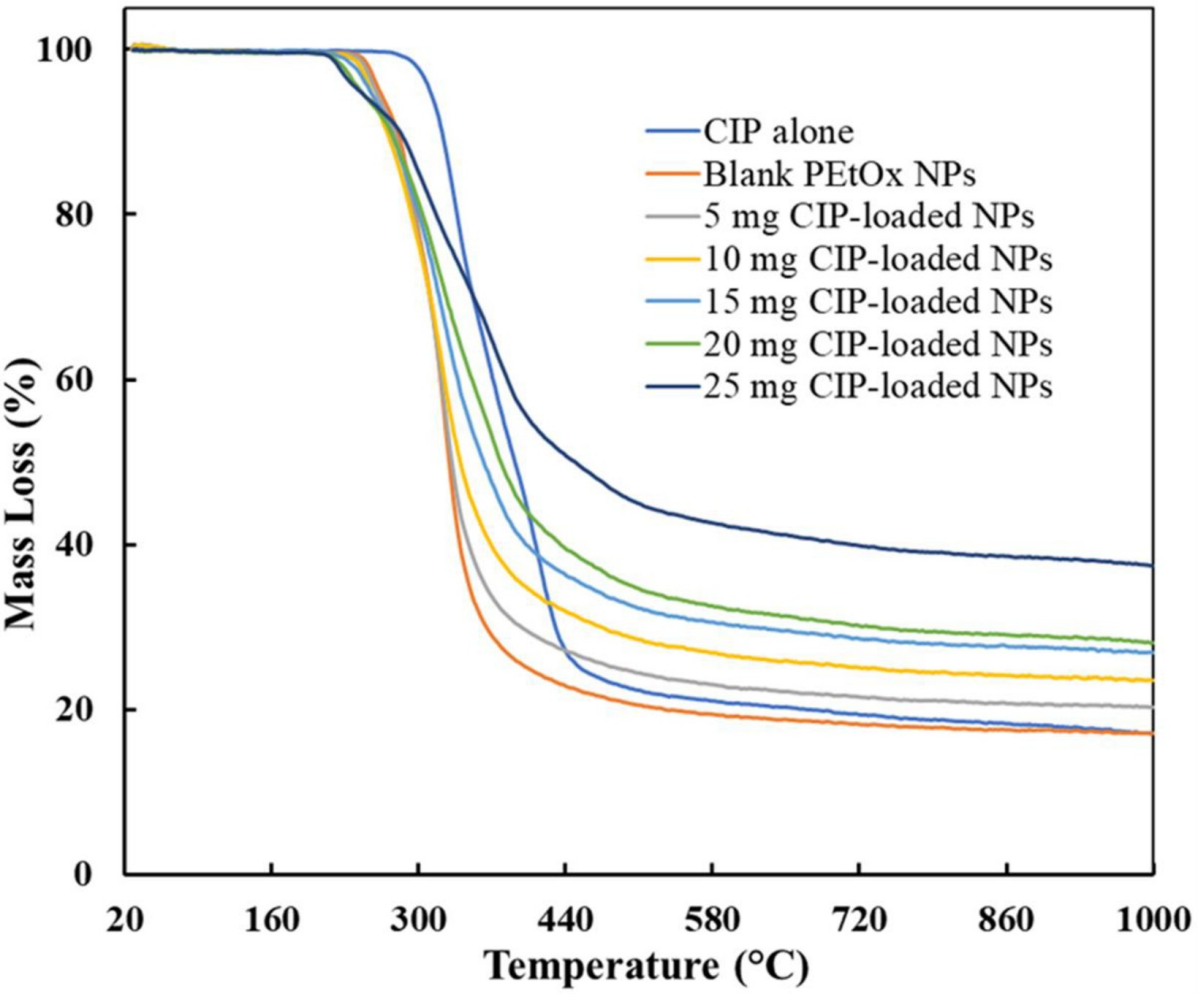
The TGA thermograms of CIP alone, blank PEtOx NPs, and different amounts of CIP-loaded PEtOx NPs.

Fig 5 represents the TGA thermograms of CIP alone, blank PEtOx NPs, and different amounts of CIP-loaded PEtOx NPs. The thermogram shows a substantial weight loss for CIP powder between 275–478°C which refers to the DSC curves at a melting point of 271.7°C (Fig 4). However, decomposition was found only in a single-stage while Sadeek et al. [39] found decomposition in two stages which perhaps occurred due to the impurity of CIP used in that study. Blank PEtOx NPs showed decomposition in between 245–365°C with 70% weight loss. However, CIP-loaded PEtOx NPs showed up to 57% weight loss until 527°C which suggests the possible interaction among PEtOx, TA, and CIP [40]. The amount of CIP in the formulations also showed crucial characteristics in the thermal behaviour of the NPs. Weight loss was increased with a decreased amount of CIP in the formulations [39]. All these characteristics of the prepared NPs indicate the thermal stability of the prepared NPs in a solid-state.

### 3.8 PXRD–crystal structure refinement

**Fig 6:**
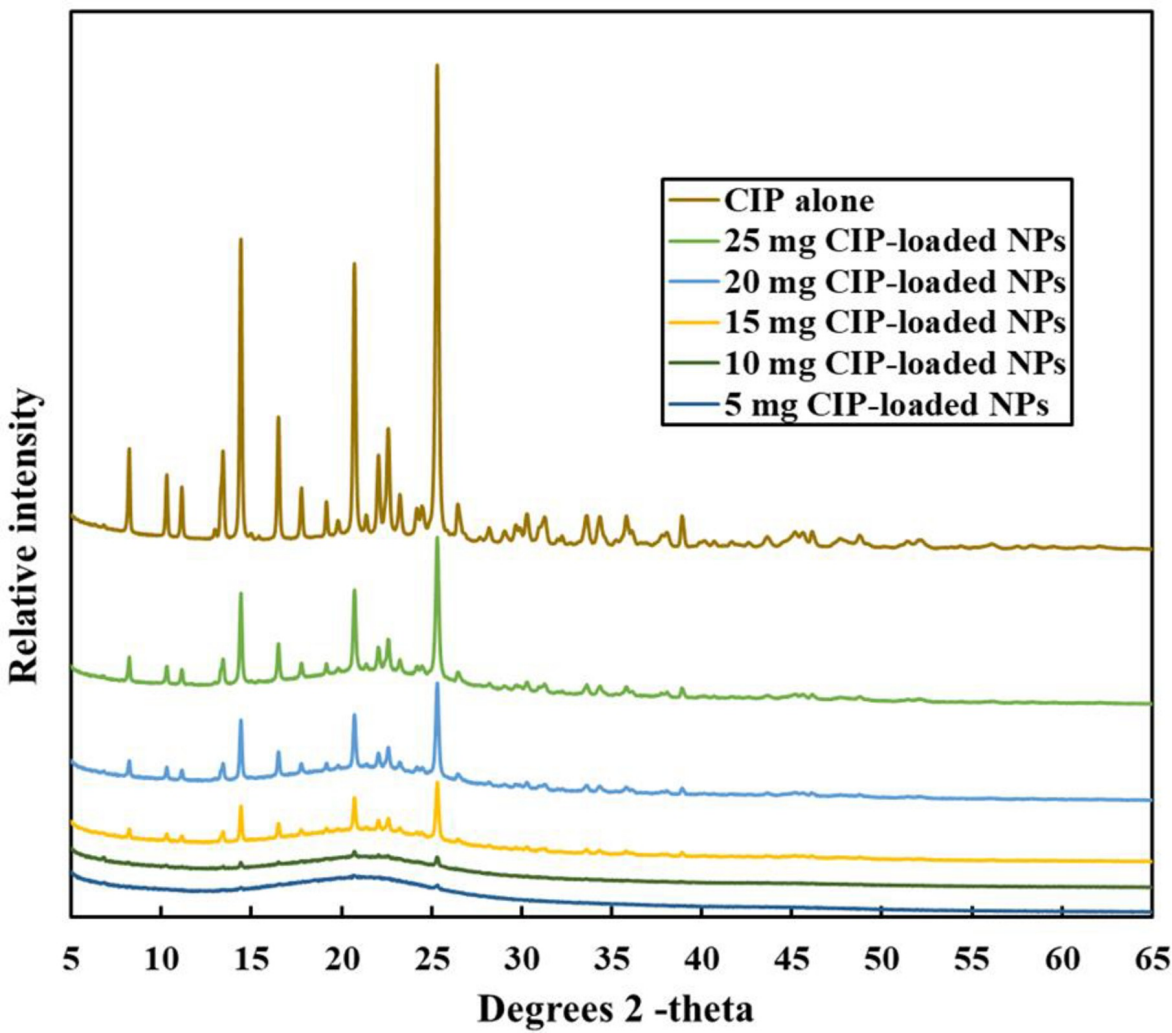
Comparison of PXRD patterns of CIP alone and different amounts of CIP-loaded PEtOx NPs.

The X-ray diffraction pattern of CIP alone and those of CIP-loaded PEtOx NPs were shown in Fig 6. CIP powder alone demonstrated three characteristic peaks at 14.42°, 20.82°, and 25.38° in the diffractogram at the angle of 2Theta as reported by others [41]. Different amounts of CIP-loaded PEtOx NPs showed the same diffraction pattern with a remarkably lower intensity with all the formulations. The presence of major characteristics intensity of CIP in the CIP-loaded PEtOx NPs suggests that CIP was present within the formulated NPs. This finding is relevant with the DSC results. The increased concentrations of drugs in the NPs showed stronger diffraction intensity peaks (Fig 6). However, 5 mg and 10 mg CIP-loaded NPs showed small traces of peaks at similar positions (Fig 6) thus suggesting a low amount of drug loading or encapsulations. Tewes et al. [42] also reported similar findings while studying CIP-loaded composite microparticles to treat a bacterial lung infection.

### 3.9 ATR-FTIR spectral analysis

**Fig 7:**
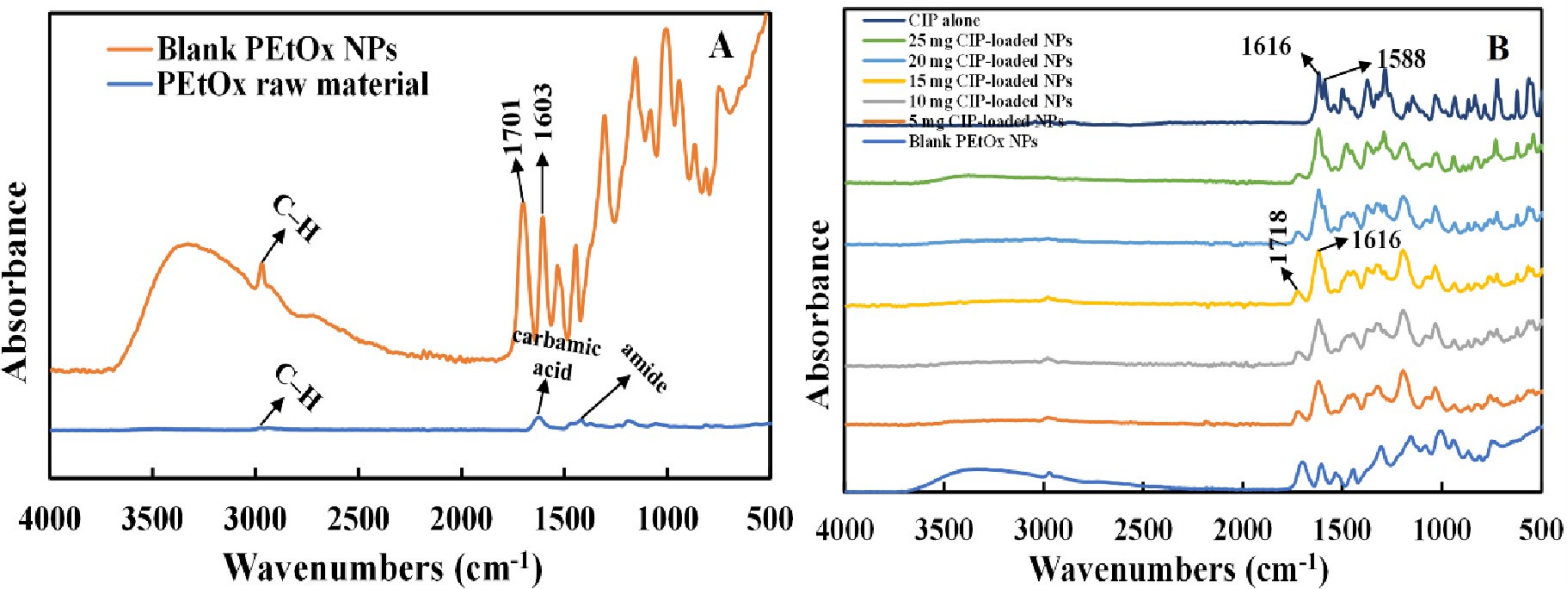
FTIR spectrum of (A) raw PEtOx and blank PEtOx NPs; (B) blank PEtOx NPs, different amounts of CIP-loaded PEtOx NPs, and CIP alone.

PEtOx raw material demonstrated several characteristic peaks in FTIR spectra (Fig 7A). C-H stretching vibration was seen at 2938 cm^-1^. Raw PEtOx showed its typical characteristic peaks at 1624 cm^-1^ and 1420 cm^-1^ which were attributed with the vibration of carbamic acid (C = O) band and amide carbonyl band, respectively. In addition, some more peaks were observed with the stretching vibration of C-H at 1371 cm^-1^ and stretching vibration of C–C at 1187 cm^-1^ and 1056 cm^-1^ which match with the previous report [14]. Blank PEtOx NPs showed their characteristic peak at 1603 cm^-1^. The shifting in lower wavenumbers occurred due to the formation of hydrogen bonding of–C = O between the polymer and the acid. Additionally, blank NPs showed a new intensive peak of C = O at 1701 cm^-1^ and confirms the formation of hydrogen bonding [43]. However, electrostatic interactions could also be the reason for NPs formation as–OH and–COOH band vibrations were still present in the blank NPs and confirms all the functionalities did not participate in hydrogen bonding [31].

Different amounts of CIP-loaded PEtOx NPs were also investigated by FTIR analysis. The obtained spectrum is presented in Fig 7B. The characteristic N–H bending vibration of quinolines was recorded at 1616 cm^-1^ and the C-H stretching vibration of alkyl groups were found at 1588 cm^-1^ for the pure CIP. CIP alone showed some more characteristic peaks at 1498 cm^-1^ and 1473 cm^-1^ which represent the C–H stretching band and match with a previous report [44]. CIP-loaded PEtOx NPs showed their characteristic peaks at 1616 cm^-1^ which corresponds to the N-H bending vibration of quinolines and confirms the presence of CIP within the formulated NPs. However, the C = O stretching vibration of the carbonyl acid group was recorded at 1718 cm^-1.^ The shifting in higher wavenumbers from the wavenumbers of the blank NPs occurred due to the formation of hydrogen bonding of–C = O among the polymer, acid, and the drug. The disappearance of the characteristic peak of the raw PEtOx polymer within the formulated CIP-loaded PEtOx NPs confirms the proper hydrogen bonding among PEtOx, CIP, and TA. Similar findings were also observed by Liu et al [31] when they used doxorubicin and poly(2-oxazoline) to formulate NPs for cancer treatment.

### 3.10 Drug loading

Drug loading and entrapment efficiency of CIP-loaded PEtOx NPs were evaluated to understand the drug loading performance of PEtOx polymer. The findings showed that increasing the amount of CIP while formulating the NPs increased the percentage of drug loading and entrapment efficiency as presented in Table 4. However, maintaining the mixing ratio of the polymer and the acid was also important to increase the drug loading.

**Table 4:** Drug loading and entrapment efficiency of CIP-loaded PEtOx NPs (data presented as mean ± S.D., n = 3).

NPs	Drug loading (%)	Entrapment efficiency (%)
**5 mg CIP-PEtOx**	21.0 ± 1.4	21.4 ± 2.0
**10 mg CIP-PEtOx**	37.5 ± 0.7	55.5 ± 2.0
**15 mg CIP-PEtOx**	43.0 ± 3.2	71.6 ± 3.2
**20 mg CIP-PEtOx**	49.6 ± 3.5	68.3 ± 5.0
**25 mg CIP-PEtOx**	67.6 ± 0.6	74.2 ± 2.5

Indirect measurements of CIP loading and entrapment efficiency were done by determining the remaining amount of CIP in the supernatant. Quantitative analysis of the drugs remaining in the supernatant was performed by UV spectrophotometry [45]. The drug loading of 5 mg to 25 mg CIP-loaded PEtOx NPs increased from 20.9 ± 1.4% to 67.6 ± 0.6% as the concentration of CIP increased. Increasing concentrations of CIP showed a significant increase (*p < 0*.05) in drug loading. A similar finding was reported by Wang et al. [7] when they formulated nicotine hydrogen tartrate-loaded chitosan NPs. However, Ajun et al. [46] found that increased concentration of drug could reach optimum drug loading value and after that increased concentration of drug could not increase the drug loading rather than decrease drug loading. Entrapment efficiency also showed to be increased significantly (*p < 0*.*05*) with the increasing amount of CIP. However, drug loading and entrapment efficiency did not show a significant relationship (*p > 0*.*05*) between them with the increasing amount of CIP. Besides, increased drug loading could also result in increased particle size according to the findings of SEM images (Fig 2) [47]. However, the prepared NPs were considerably smaller in size, thus, it is obvious that the maximum entrapment efficacy was achieved in this study.

### 3.11 *In vitro* controlled release study of CIP from CIP-loaded PEtOx NPs

**Fig 8:**
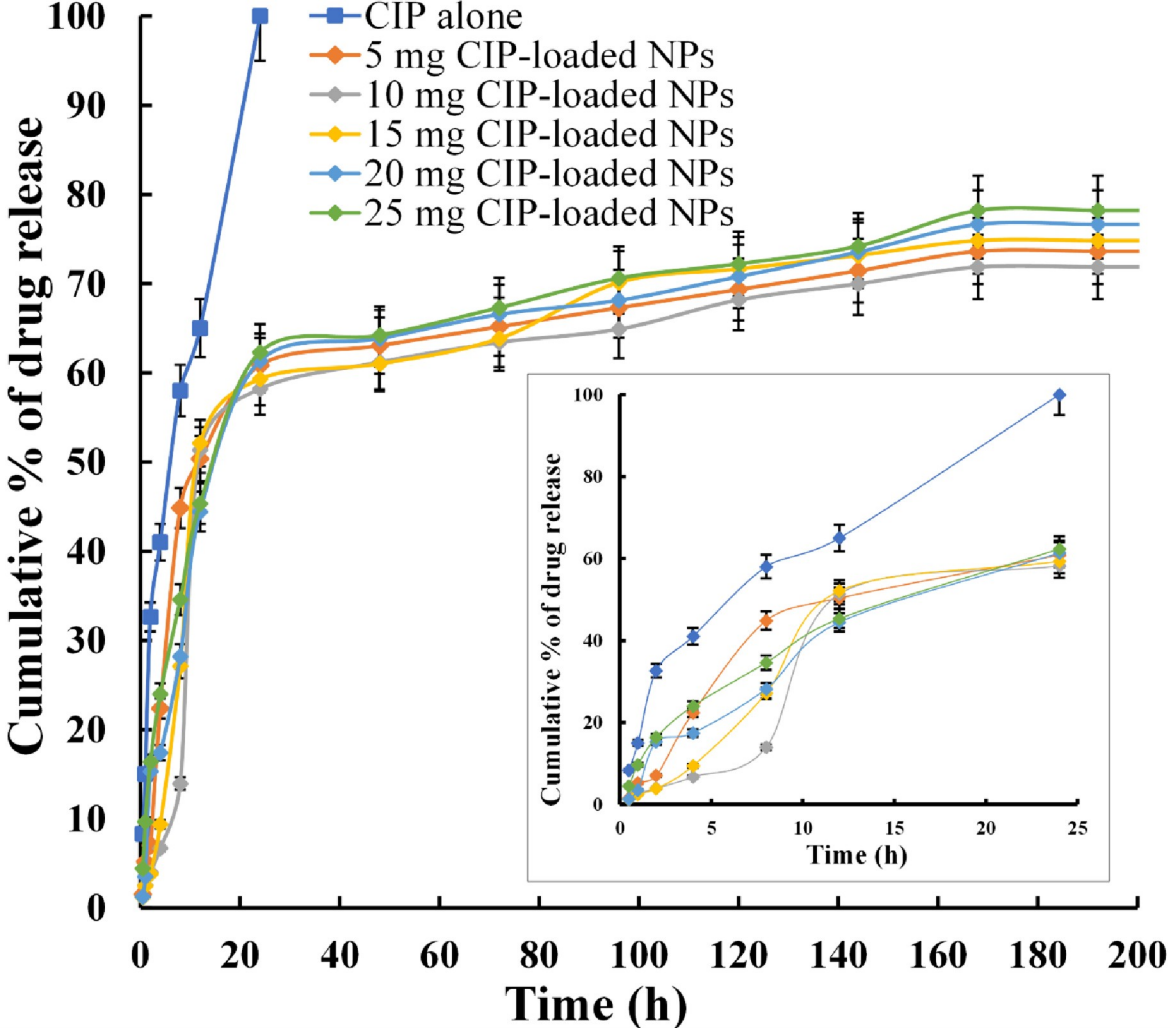
*In vitro* release profile of CIP from CIP-loaded PEtOx NPs for 14 days and release profile for first 24 hours in the bottom right (data presented as mean ± S.D., n = 3).

The cumulative percentage of CIP release from CIP-loaded PEtOx NPs was presented in Fig 8. Pure CIP showed 100% diffusion through the dialysis membrane bag within the first 24 hours in the *in vitro* release study, thus, it was expected that CIP-loaded PEtOx NPs could effectively extend the release of CIP. As demonstrated in Fig 8 all the formulations achieved maximum cumulative % release after seven days. 5 mg CIP-loaded PEtOx NPs achieved a maximum of 73.5% cumulative release with an initial 50.8% release in the first 12 hours. Meantime, 10 mg, and 15 mg CIP-loaded PEtOx NPs achieved a maximum of 71.9% and 74.8% cumulative release with 51.2% and 52.1% initial burst release, respectively. Consequently, 20 mg, and 25 mg CIP-loaded PEtOx NPs achieved a maximum 76.6% and 78.2% cumulative release with 44.4% and 45.3% initial burst release, respectively. These findings indicate that an increased amount of CIP in the formulations provided more CIP release from CIP-loaded PEtOx NPs [27]. Moreover, the aerosolization studies also revealed that high dispersion could be achieved with increased drug loading. Thus, high dispersion behaviour within the particles caused rapid drug release from the NPs at a longer time. Besides, the particle size of the NPs also played important roles in the initial and maximum cumulative release of CIP from the polymeric NPs. Similar findings were also reported by Wang et al. [7] when they formulated chitosan-based hydrophilic drug-loaded NPs. However, the finding also reveals that prolonged drug release is more dependent on drug loading rather than the surface properties of the NPs. Meantime, the delayed maximum cumulative % release of drug (7 days) from the CIP-loaded PEtOx NPs compared to CIP alone release occurred due to the release of CIP from the inner core of the fused NPs. In addition, slow initial burst release from the NPs confirms that the drugs were properly entrapped within the matrix of the NPs rather than the outer surface [48]. At the end of the release study, the maximum cumulative % release was about 78% which was more than the previous report by Gharrari et al. [49] while studying CIP-loaded alginate/chitosan NPs. This indicates that hydrogen bonding is more suitable compared to other formulation techniques of NPs preparation to get maximum cumulative % release. Our experimentation was continued for 14 days, and CIP release from CIP-loaded PEtOx NPs was still noticed, thus, CIP release from CIP-loaded PEtOx NPs was not finished. Therefore, further studies with a longer duration could be done to get a complete understanding of CIP release from CIP-loaded PEtOx NPs.

Our findings showed that the CIP release pattern from the CIP-loaded PEtOx NPs was extensively controlled by the amount of CIP present in the NPs and the surface area of the NPs. However, initial drug release was comparatively low compared to CIP-loaded alginate/chitosan formulations while it was studied for understanding the controlled release behaviour of CIP [50]. Initial burst release could be modified by changing the molecular weights of the polymers while formulating [48]. Thus, further studies are warranted to utilize different molecular weights PEtOx while formulating CIP-loaded PEtOx NPs to understand the complete initial burst release characteristics of the formulations.

To the best of our knowledge, PEtOx polymer has never been studied for lung delivery and its drug release behaviour has not been determined yet especially for DPI formulations. We studied PEtOx polymer for the first time to determine its suitability in pulmonary delivery and understand its controlled release behaviour as DPI formulations. However, the biocompatibility of PEtOx polymer and its NPs in bronchial epithelial cell lines and their degradability behaviour in lung fluids still need to be determined.

### 3.12 Kinetics of drug release

Our knowledge is limited on drug release kinetics, especially for inhalable drugs. Till now, the metabolic fate of lung delivery for CIP-loaded PEtOx NPs has not been investigated. The drug release rate from polymer-based formulations depends on the diffusion pattern of the formulated drugs in liquid media and the degradation mechanism of the polymer. The *in vitro* drug release data of CIP-loaded PEtOx NPs was fitted into different kinetics models of drug release (Table 5) to get an understanding of the CIP release mechanism from PEtOx polymer. The best fitted model was selected based on the highest values of r^2^. According to the zero-order model drug release remains constant throughout the delivery system thus maintaining a constant drug blood level. The release data (Table 5) revealed that all the formulations were poorly fitted with the first-order model thus drug release was not constant from the CIP-loaded PEtOx NPs. Hixson-Crowell model was also ruled out as the data showed poorly fittings with the model thus dissolution-type release kinetics is not suitable to assume the pattern of the drug release [51]. The first-order release kinetics model is related to the concentration of the drug, the faster the concentration of drug increases in a delivery system the faster the drug release occurs. The release data (Table 5) indicates that 20 mg and 25 mg CIP-loaded PEtOx NPs compared to the other three formulations fit more with the first order and confirms our previous statement that the higher the drug concentration the more the maximum cumulative % of drug release is achieved. However, this model is not a good fit as other formulations are poorly fitted with this model. Similarly, increased amounts of CIP in 20 mg and 25 mg CIP-loaded PEtOx NPs followed the Higuchi model, however, 5 mg CIP-loaded PEtOx NPs did not fit well with this model (r^2^ = 0.7789). Thus, diffusion could be the possible mechanism of drug release from the CIP-loaded PEtOx NPs. However, there are also possibilities of a slower diffusion rate compared to polymer degradation. Similar findings were also reported by Wang et al. [7] while studying the release profile of nicotine hydrogen tartrate loaded in chitosan NPs. Finally, the drug release data were fitted into the semiempirical Korsmeyer-Peppas model [24]. All the release data from the CIP-loaded PEtOx NPs fitted with this model (r^2^ = 0.835–0.9096), thus release exponent (*n*) was determined to understand more about the release mechanism. A curve was plotted using the cumulative log% data versus log time to calculate the release exponent. This release exponent (*n*) value is considered to understand the release mechanisms from various types of drug delivery systems. Theoretically, the *n* value is considered to determine whether the release mechanism follows the Fickian diffusion mechanism or the non-Fickian anomalous transport mechanism. According to the Fickian diffusion, the drug moves from high molecular concentration regions to low molecular concentration regions using a diffusion mechanism [52]. According to non-Fickian transport both diffusion and polymer degradation combinedly control the release mechanism. If *n* is 0.45 or less then it follows Fickian diffusion, if it’s between 0.45 to 0.89 then it follows non-Fickian transport. However, if the *n* value is more than 0.89 then drug release can occur by polymer degradation and polymeric chain erosion with a constant drug release rate [53]. The findings showed *n* values for all the formulations except 25 mg CIP-loaded PEtOx NPs were between 0.45 and 0.89 (0.5756–0.7074) indicating the drug release might follow non-Fickian or anomalous transport thus suggesting drug release was regulated by both diffusion and polymer degradation. The findings of polymer degradation also suggest that there is a possibility of PEtOx degradation in lung fluids as the release medium resembles the pH of lung fluids. In contrast, the *n* value of 25 mg CIP-loaded PEtOx was less than 0.45 (0.4368), which indicates to follow Fickian diffusion thus confirming the outer surface of the NPs was covered with more CIP molecules and preventing the degradation of the polymer.

**Table 5:** Kinetics of CIP release from CIP-loaded PEtOx NPs.

Kinetics model	5 mg CIP-loaded NPs	10 mg CIP-loaded NPs	15 mg CIP-loaded NPs	20 mg CIP-loaded NPs	25 mg CIP-loaded NPs
**Zero-order (*F* = *k***_**0**_ ***t*)**					
***k***_**0**_	0.3537	0.4003	0.4072	0.3908	0.3725
**r** ^**2**^	0.5995	0.6444	0.6585	0.6836	0.6923
**First-order (ln(1−*F*) = −*k***_**1**_ ***t*)**					
***k***_**1**_	0.003	0.0032	0.0034	0.0034	0.0034
**r** ^**2**^	0.7264	0.7396	0.779	0.8018	0.8172
**Higuchi** $\left(F=k_{H}t^{\frac{1}{2}}\right)$					
***k***_***H***_	5.4318	6.041	6.145	5.8807	5.5959
**r** ^**2**^	0.7789	0.8087	0.8264	0.8528	0.8609
**Hixson-Crowell** $\left(1-{\left(1-F\right)}^{\frac{1}{3}}=k_{\frac{1}{3}}t\right)$					
$k_{\frac{1}{3}}$	0.0085	0.0091	0.0097	0.0095	0.0094
**r** ^**2**^	0.6829	0.7058	0.7377	0.7623	0.7762
**Korsmeyer-Peppas (*F* = *K***_***k*−*p***_ ***t*** ^***n***^ **)**					
***k***	8.216	8.681	8.3912	6.0215	5.6124
**n**	0.5756	0.7074	0.7025	0.5997	0.4368
**r** ^**2**^	0.8351	0.9077	0.8917	0.8511	0.9096

A variety of factors such as CIP/PEtOx concentrations and molecular weight of PEtOx, the concentration of co-assembler, particle size and degradation of PEtOx polymer control the CIP release mechanism from CIP-loaded PEtOx NPs. Thus, a complex process was involved in the release mechanism of CIP from the NPs. However, the *in vitro* experimentations confirm that neutral polymer PEtOx could be used as a carrier to develop CIP-loaded NPs. It is expected that this could provide controlled release behaviour over CIP for pulmonary delivery.

### 3.13 *In vitro* aerosolization study

The flow property of blank PEtOx NPs and CIP-loaded PEtOx NPs were evaluated in terms of RD, ED, FPF, and FPD and presented in Table 6. All formulations provided the percentages of RD in between 94% to 98% (Table 6), suggesting particles were properly dispersed from the mouthpiece and various stages of TSI. However, the particle surface charge could have been the reason for attaching with the glass surface of the inhaler device and could not disperse the rest of the particles. The ED of the NPs was between 83.1% to 89%. Significant difference (*p* < 0.05) was observed between the ED of blank PEtOx NPs and CIP-loaded PEtOx NPs (86.0 ± 1.4% vs 89.0 ±1.3%). The FPFs of all the NPs were between 35% to 41% (Table 6). The blank PEtOx NPs showed maximum FPF (41.0 ± 1.1%); however, no significant differences among the FPF of blank PEtOx NPs and CIP-loaded PEtOx NPs containing 20 and 25 mg of CIP were observed (*p* > 0.05) because of higher drug loading in those NPs. As the blank PEtOx NPs showed a higher negative charge compared to CIP-loaded PEtOx NPs thus it could play role in exhibiting higher repulsive force that resulted in higher FPF [54]. Regarding the FPFs of CIP loaded NPs (Table 6), no significant differences (*p* < 0.05) were observed among 5, 10 and 15 mg formulations; however, significantly lower FPF of 15 mg formulation was found compared to those of 20 (p = 0.003) and 25 mg (p = 0.001) formulations. As discussed earlier, CIP-loaded PEtOx showed a lower negative charge as the surface negative charge was covered by the CIP molecules thus reducing the deagglomerates characteristics compared to the blank PEtOx NPs. This resulted in low FPF of CIP loaded NPs compared to that of blank PEtOx NPs. As drug-loaded NPs showed the increased size and surface roughness compared to blank PEtOx NPs, a fixed amount of formulations might accommodate less number of particles thus causing to reduce the FPF [7].

**Table 6:** *In vitro* evaluation of particle deposition from blank PEtOx NPs and CIP-loaded PEtOx NPs (n = 5; data presented as mean ± S.D.).

Formulations	RD (%)	ED (%)	FPF (%)	FPD (mg)
**Blank PEtOx**	98.8 ± 1.0	86.0 ± 1.4	41.0 ± 1.1	-
**5 mg CIP-PEtOx**	94.8 ± 1.8	83.1 ± 0.9	36.8 ± 0.6	0.7 ± 0.1
**10 mg CIP-PEtOx**	95.3 ± 2.1	87.0 ± 1.6	37.0 ± 0.5	2.0 ± 0.1
**15 mg CIP-PEtOx**	96.6 ± 4.0	86.5 ± 0.4	35.0 ± 0.7	2.1 ± 0.2
**20 mg CIP-PEtOx**	96.9 ± 1.0	89.0 ± 1.3	38.1 ± 1.0	2.9 ± 0.1
**25 mg CIP-PEtOx**	97.1 ± 3.5	87.7 ± 0.7	39.1 ± 0.4	4.5 ± 0.1

CIP-loaded PEtOx NPs showed increased FPF with the increased CIP loading within the formulations (Table 6). Some physiochemical factors or surface properties play important roles in the successful dispersion of particles, including mass median aerodynamic diameter (MMAD), surface smoothness, electrostatic forces among the particles, and the Vander Waals forces [55]. According to the SEM images, surface roughness was increased with the increased drug loading in case of CIP-loaded PEtOx NPs (Fig 2F). Thus, surface roughness was increased with the increasing amount of CIP in the formulations which resulted in less contact and cohesion forces among the NPs thus increasing FPF [7]. However, 15 mg CIP-loaded PEtOx NPs showed significantly (*p* < 0.003) lower FPF compared to 20 and 25 mg CIP-loaded PEtOx NPs because of the larger particle size (Table 2), which have lower surface area associated with lower amount of drug compared to those of smaller particles [34]. In addition, the physicochemical properties (Table 3) also played key roles in increasing FPF of CIP-loaded PEtOx NPs containing 20 and 25 mg CIP in those NPs. All the flow property parameters including Carr’s index (CI), Hausner ratio (HR), and angle of repose (θ) showed decreasing trends with increasing drug loading. As discussed earlier increased drug loading resulted in increased particle sizes and thus improves the dispersibility behaviour of larger particles, therefore, increased the FPF.

The amount of CIP was deposited into the stage 2 of the TSI was between 0.6 mg to 4.5 mg (FPD, Table 6). Based on our experimental data, it is anticipated that the above-mentioned amounts of drugs would be delivered into the deep regions of the lung. According to the drug release data, 2.025 mg CIP would be released in the first 12 hours and the maximum amount of drug (4.5 mg, Table 6) would be achieved from 25 mg CIP-loaded PEtOx NPs in 7 days. It has been reported that the plasma concentration of 2.3 mg/L was achieved from 200 mg intravenous infusion [56]. In our study, the calculated FPD (2.03 mg) of the 25 mg CIP-loaded PEtOx NPs (one capsule) deposited in the deep lungs is assumed to be absorbed from the lungs after inhalation. Thus, the desired therapeutic concentration of 2.3 mg/L (intravenous administration) in plasma could be achieved by approximately 40 mg of the prepared NPs DPI formulation (i.e., two capsules). However, further studies using animal model are warranted to get a complete understanding of the therapeutic concentration of the CIP from drug-loaded PEtOx NPs. Overall, this study demonstrated that hydrogen bonding between hydrogen donor TA and acceptor PEtOx could be utilized to formulate CIP-loaded PEtOx NPs for lung delivery from DPI formulations. It is expected that the safety profile of PEtOx polymer for lung delivery could be achieved by further investigations in animal models. The findings from this study might be helpful to formulate PEtOx NPs using other drugs and could be helpful to achieve a more sustained release behaviour potential polymer-based formulation.

## 4. Conclusion

This study demonstrated an effective and feasible way to develop inhalable CIP-loaded PEtOx NPs for pulmonary delivery against LRTIs. Hydrogen bonding between the neutral polymer PEtOx and weak acid TA played key roles in preparing CIP-loaded PEtOx NPs. The drug CIP did not show any interactions with PEtOx as confirmed by ATR-FTIR, PXRD, DSC, and TGA analysis. More than 67% CIP was loaded in this polymer and the loading of CIP in PEtOx was CIP concentration-dependent i.e., the higher the drug concentration, the higher the drug loading. The CIP-loaded PEtOx NPs showed an extended drug release profile (for 168 h) at maximum drug loading (67.6%). The kinetics of CIP release followed non-Fickian or anomalous transport, which suggested that CIP release was controlled by both diffusion and polymer degradation. However, further studies are warranted to investigate the degradation of PEtOx in lung fluids. FPF of CIP ranging from 36.4–39.1% showed that this drug could be delivered within the deep lungs up to 39% from the PEtOx polymer carrier. The outcome of this study revealed the potential of CIP-loaded PEtOx NPs for pulmonary drug delivery to combat the LRTIs with potential therapeutic outcomes; however, further in-vivo investigations are warranted to understand the suitability of this polymer for lung delivery. This study also opens a window to introduce a new polymer PEtOx as a carrier in lung drug delivery and it can be used to formulate other drugs.

## Supporting information

S1 File (PDF)
