# Supplementary material for: Inhaled ciprofloxacin-loaded poly(2-ethyl-2-oxazoline) nanoparticles from dry powder inhaler formulation for the potential treatment of lower respiratory tract infections
Source: PLoS One. 2021 Dec 23;16(12):e0261720. doi: 10.1371/journal.pone.0261720 (PMC8699692; doi:10.1371/journal.pone.0261720)
Supplement: S1 File — (PDF) [file pone.0261720.s001.pdf]

Supporting Information

The crystal structure of ciprofloxacin used in this study from Rietveld refinement:

```
data_
_chemical_name_mineral 1-Cyclopropyl-6-fluoro-4-oxo-7-(piperazin-4-ium-1-yl)-1,4-dihydroquinoline-3-
carboxylate
_cell_length_a 7.96045562
_cell_length_b 8.57791746
_cell_length_c 10.7735382
_cell_angle_alpha 87.8568055
_cell_angle_beta 85.1422169
_cell_angle_gamma 88.2246609
_cell_volume 732.220576
_symmetry_space_group_name_H-M P-1
loop_
_symmetry_equiv_pos_as_xyz
'x, y, z '
'-x, -y, -z '
loop_
_atom_site_label
_atom_site_type_symbol
_atom_site_site_symmetry_multiplicity
_atom_site_fract_x
_atom_site_fract_y
_atom_site_fract_z
_atom_site_occupancy
_atom_site_B_iso_or_equiv
F_1 F   2 1.1755(8) 0.0347(13) -0.2586(11) 1 1.32647
O_1 O   2 1.322(5) 0.432(5) 0.414(3) 1 1.32647
O_2 O   2 1.437(4) 0.197(5) 0.389(4) 1 1.32647
O_3 O   2 1.449(2) 0.149(3) 0.1245(18) 1 1.32647
N_1 N   2 0.959(2) 0.3177(18) 0.1797(8) 1 1.32647
N_2 N   2 0.8439(7) 0.1566(7) -0.2351(5) 1 1.32647
N_3 N   2 0.6215(5) 0.2382(4) -0.4289(3) 1 1.32647
C_1 C   2 0.781(3) 0.351(3) 0.2156(6) 1 1.32647
C_2 C   2 0.728(4) 0.500(3) 0.2779(19) 1 1.32647
C_3 C   2 0.711(3) 0.482(3) 0.1402(9) 1 1.32647
C_4 C   2 1.0101(19) 0.2487(16) 0.0693(8) 1 1.32647
C_5 C   2 1.067(3) 0.328(2) 0.2683(10) 1 1.32647
C_6 C   2 1.231(3) 0.273(3) 0.2560(14) 1 1.32647
C_7 C   2 1.328(3) 0.301(3) 0.3652(16) 1 1.32647
C_8 C   2 1.298(2) 0.200(2) 0.1436(15) 1 1.32647
C_9 C   2 1.1808(18) 0.1881(19) 0.0487(12) 1 1.32647
C_10 C   2 1.2331(13) 0.1176(18) -0.0644(13) 1 1.32647
C_11 C   2 1.1220(10) 0.1080(14) -0.1531(10) 1 1.32647
C_12 C   2 0.9508(10) 0.1662(11) -0.1382(6) 1 1.32647
C_13 C   2 0.6732(8) 0.2206(8) -0.2031(9) 1 1.32647
C_14 C   2 0.5543(8) 0.1867(5) -0.3011(8) 1 1.32647
C_15 C   2 0.7961(11) 0.1751(6) -0.4594(11) 1 1.32647
C_16 C   2 0.9092(8) 0.2207(7) -0.3603(13) 1 1.32647
C_17 C   2 0.8988(15) 0.2358(12) -0.0249(6) 1 1.32647
H_1 H   2 0.701(3) 0.266(3) 0.2395(13) 1 1.58703
H_2 H   2 0.817(4) 0.567(3) 0.303(3) 1 1.58703
H_3 H   2 0.624(4) 0.499(3) 0.335(2) 1 1.58703
H_4 H   2 0.791(4) 0.536(3) 0.0784(12) 1 1.58703
H_5 H   2 0.595(4) 0.470(3) 0.1134(16) 1 1.58703
H_6 H   2 1.028(3) 0.376(2) 0.3433(10) 1 1.58703
H_7 H   2 1.3457(13) 0.077(2) -0.0789(15) 1 1.58703
H_8 H   2 0.6293(9) 0.1757(10) -0.1211(9) 1 1.58703
H_9 H   2 0.6784(8) 0.3340(8) -0.1955(8) 1 1.58703
H_10 H   2 0.5341(10) 0.0733(6) -0.2999(9) 1 1.58703
H_11 H   2 0.4456(8) 0.2416(7) -0.2805(6) 1 1.58703
H_12 H   2 0.8405(13) 0.2167(7) -0.5418(11) 1 1.58703
H_13 H   2 0.7952(13) 0.0597(6) -0.4634(13) 1 1.58703
H_14 H   2 1.0256(9) 0.1807(9) -0.3820(15) 1 1.58703
```

```
H_15 H   2 0.9129(8) 0.3360(8) -0.3588(11) 1 1.58703
H_16 H   2 0.7857(16) 0.2751(11) -0.0111(4) 1 1.58703
H_17 H   2 0.6233(9) 0.3378(5) -0.4344(7) 1 1.58703
H_18 H   2 0.5630(12) 0.2087(7) -0.4881(7) 1 1.58703
```

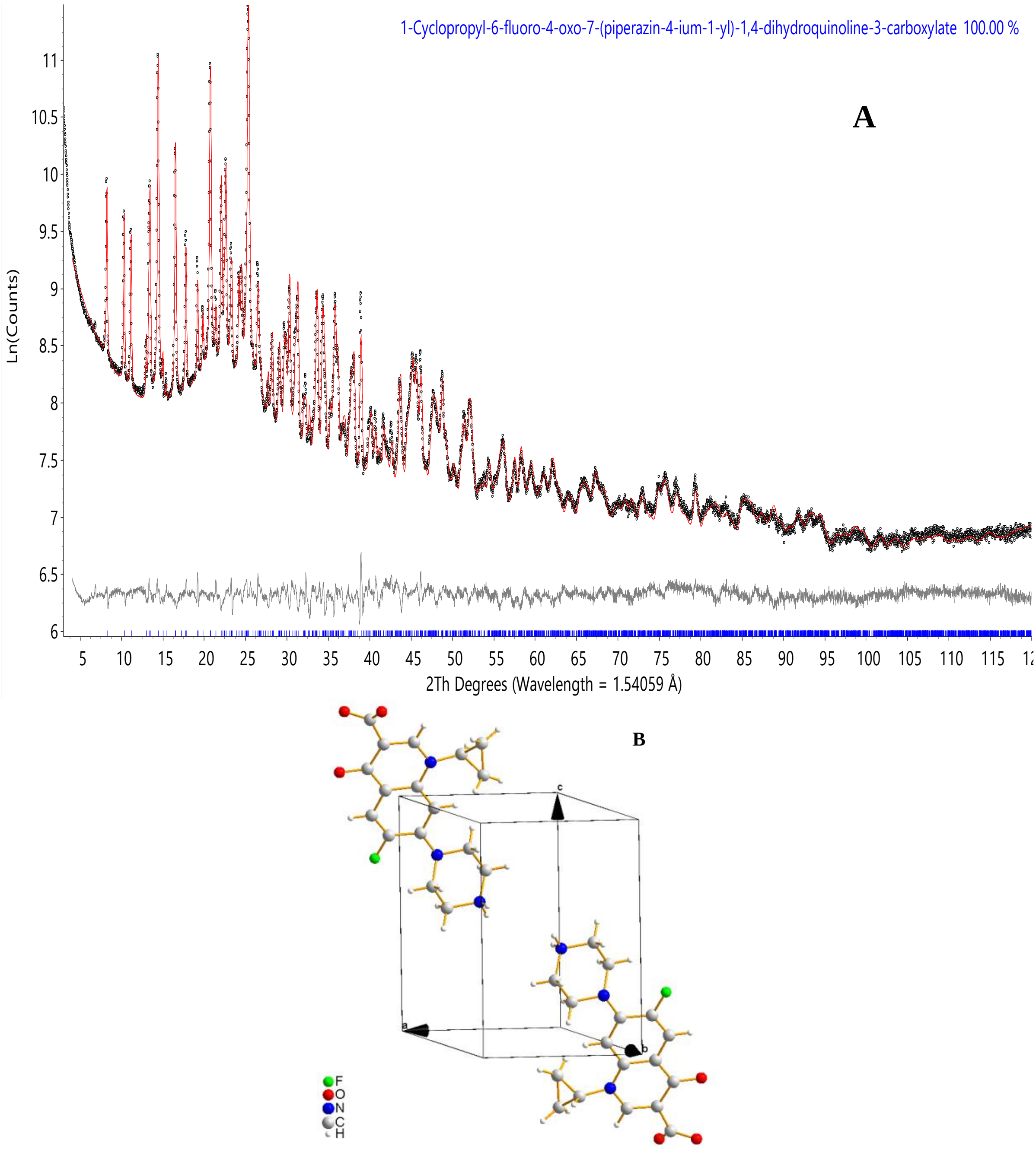

Fig S1. The fitting of XRPD pattern for Qualitative phase analysis of (A) the Rietveld refinement using rigid body model of CIP to the measured PXRD patterns of the API phase, Rwp = 5.71%, GOF = 3.04; (B) the refined crystal structure of the API phase CIP used in this study. All the XRD patterns in this paper are shown on a logarithmic scale to emphasize minor peaks.

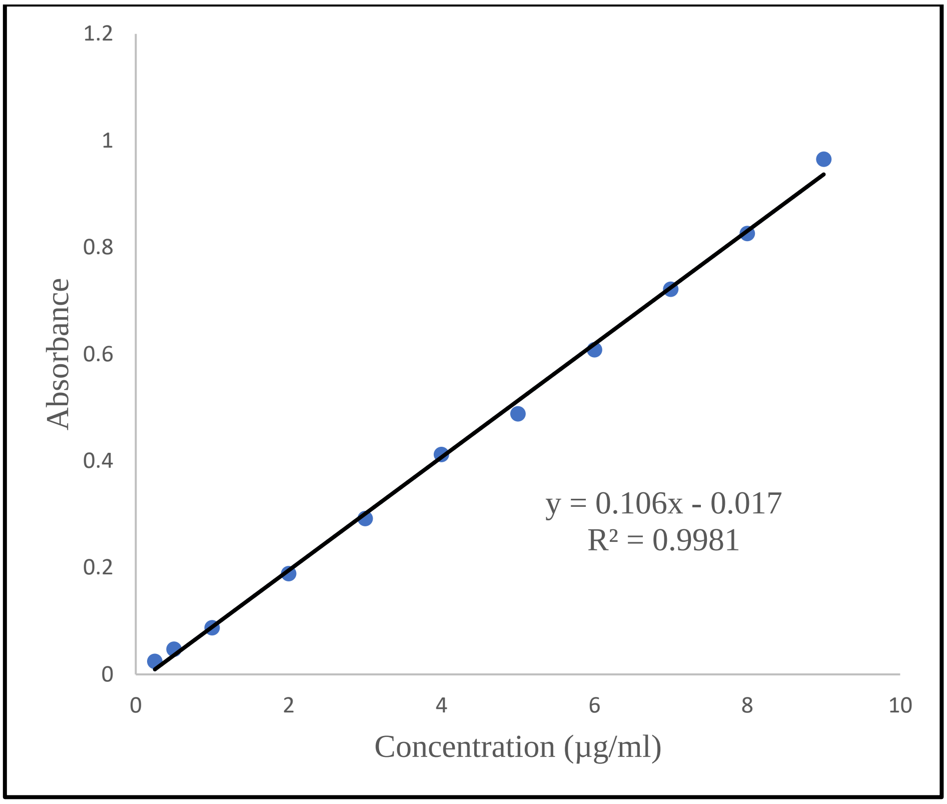

Fig S2. Calibration plot of CIP in PBS (pH 7.4) solution at the concentration range of 1 µg/ml to 9 µg/ml

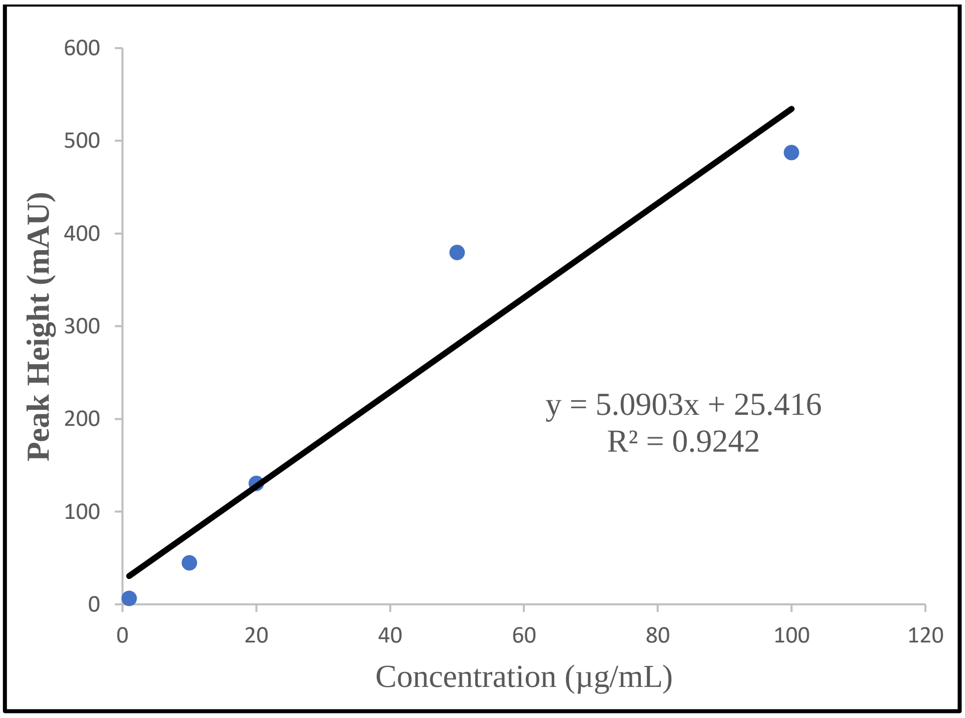

Fig S3. Calibration plot of CIP in PBS (pH 7.4) solution at the concentration range of 1 µg/ml to 100 µg/ml

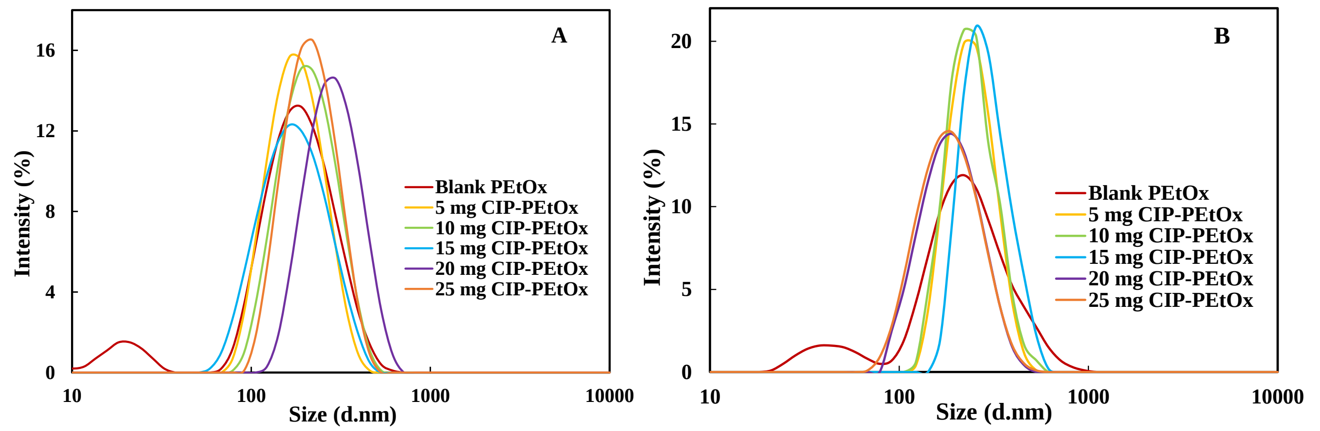

Fig S4. DLS particle size distribution of blank PEtOx NPs and different amounts of CIP-loaded PEtOx NPs from 5-25 mg (A) before freeze-drying; (B) after freeze-drying (n = 3)

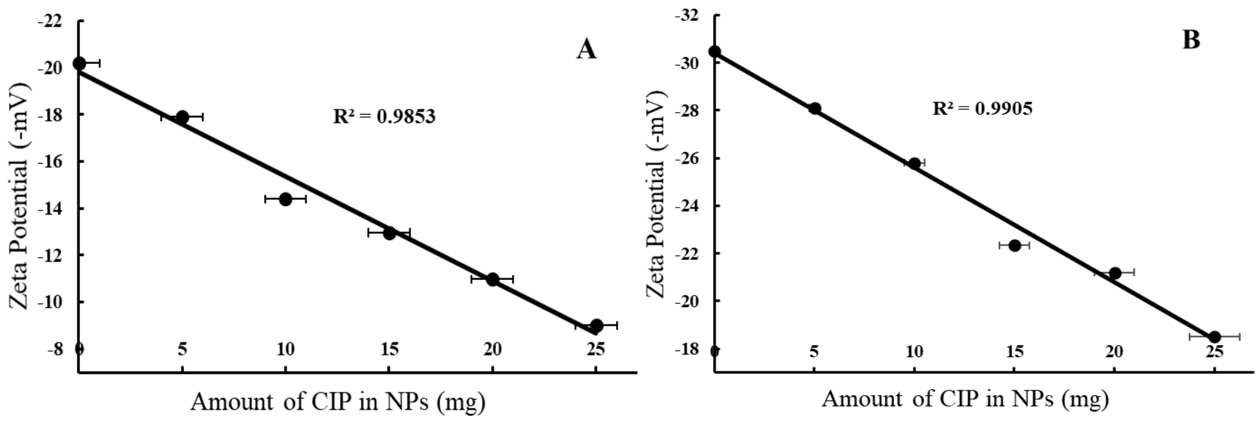

Fig S5. Surface charge relationship between blank PEtOx NPs and different amounts of CIP-loaded PEtOx NPs (A) before freeze-drying; (B) after freeze-drying (n = 3)
